# Pharmacodynamics of atabecestat (JNJ-54861911), an oral BACE1 inhibitor in patients with early Alzheimer’s disease: randomized, double-blind, placebo-controlled study

**DOI:** 10.1186/s13195-018-0415-6

**Published:** 2018-08-23

**Authors:** Maarten Timmers, Johannes Rolf Streffer, Alberto Russu, Yushin Tominaga, Hiroko Shimizu, Ayako Shiraishi, Kanaka Tatikola, Pascale Smekens, Anne Börjesson-Hanson, Niels Andreasen, Jorge Matias-Guiu, Miquel Baquero, Mercè Boada, Ina Tesseur, Luc Tritsmans, Luc Van Nueten, Sebastiaan Engelborghs

**Affiliations:** 1 Janssen Research and Development, a Division of Janssen Pharmaceutica NV, Turnhoutseweg 30, 2340 Beerse, Belgium; 2 Reference Center for Biological Markers of Dementia (BIODEM), Institute Born-Bunge, University of Antwerp, Antwerp, Belgium; 3 Janssen Pharmaceutical K.K, Tokyo, Japan; 4 Janssen Research and Development LLC, Raritan, NJ USA; 5 Sahlgrenska University Hospital, Mölndal, Sweden; 6 Department Neurobiology, Care Sciences & Society, Center for Alzheimer Research, Division of Neurogeriatrics, Karolinska Institutet, Stockholm, Sweden; 7 Hospital Clinic San Carlos, Universidad Complutense, Madrid, Spain; 8 Neurology Department, Hospital Universitari I Politecnic La Fe, Valencia, Spain; 9 Fundació ACE, Institut Català de Neurociències Aplicades, Barcelona, Spain; 10 Department of Neurology and Memory Clinic, Hospital Network Antwerp (ZNA) Middelheim and Hoge Beuken, Antwerp, Belgium; 11 Present address: Head of Translational Medicine Neuroscience, UCB Biopharma SPRL, Chemin du Foriest, B-1420 Braine-l’Alleud, Belgium

**Keywords:** Atabecestat, JNJ-54861911, BACE1 inhibitor, Alzheimer’s disease, Amyloid, Aβ processing, PK/PD relationship

## Abstract

**Background:**

β-Secretase enzyme (BACE) inhibition has been proposed as a priority treatment mechanism for Alzheimer’s disease (AD), but treatment initiation may need to be very early. We present proof of mechanism of atabecestat (also known as JNJ-54861911), an oral BACE inhibitor for the treatment of AD, in Caucasian and Japanese populations with early AD who do not show signs of dementia.

**Methods:**

In two similarly designed phase I studies, a sample of amyloid-positive elderly patients comprising 45 Caucasian patients with early AD diagnosed as preclinical AD (*n* = 15, Clinical Dementia Rating [CDR] = 0) or with mild cognitive impairment due to AD (*n* = 30, CDR = 0.5) and 18 Japanese patients diagnosed as preclinical AD (CDR-J = 0) were randomized 1:1:1 to atabecestat 10 or 50 mg or placebo (*n* = 6–8/treatment) daily for 4 weeks. Safety, pharmacokinetics (PK), and pharmacodynamics (PD) (i.e., reduction of cerebrospinal fluid [CSF] amyloid beta 1–40 [Aβ_1–40_] levels [primary endpoint] and effect on other AD biomarkers) of atabecestat were evaluated.

**Results:**

In both populations, atabecestat was well tolerated and characterized by linear PK and high central nervous system penetrance of unbound drug. Atabecestat significantly reduced CSF Aβ_1–40_ levels from baseline at day 28 in both the 10-mg (67–68%) and 50-mg (87–90%) dose groups compared with placebo. For Caucasians with early AD, the least squares mean differences (95% CI) were − 69.37 (− 72.25; − 61.50) and − 92.74 (− 100.08; − 85.39), and for Japanese with preclinical AD, they were − 62.48 (− 78.32; − 46.64) and − 80.81 (− 96.13; − 65.49), respectively. PK/PD model simulations confirmed that once-daily 10 mg and 50 mg atabecestat can attain 60–70% and 90% Aβ_1–40_ reductions, respectively. The trend of the reduction was similar across the Aβ_1–37_, Aβ_1–38_, and Aβ_1–42_ fragments in both atabecestat dose groups, consistent with Aβ_1–40_. CSF amyloid precursor protein fragment (sAPPβ) levels declined from baseline, regardless of patient population, whereas CSF sAPPα levels increased compared with placebo. There were no relevant changes in either CSF total tau or phosphorylated tau 181P over a 4-week treatment period.

**Conclusions:**

JNJ-54861911 at 10 and 50 mg daily doses after 4 weeks resulted in mean CSF Aβ_1–40_ reductions of 67% and up to 90% in both Caucasian and Japanese patients with early stage AD, confirming results in healthy elderly adults.

**Trial registration:**

ALZ1005: ClinicalTrials.gov, NCT01978548. Registered on 7 November 2013.

ALZ1008: ClinicalTrials.gov, NCT02360657. Registered on 10 February 2015.

**Electronic supplementary material:**

The online version of this article (10.1186/s13195-018-0415-6) contains supplementary material, which is available to authorized users.

## Background

β-Secretase enzyme (BACE) inhibition has been proposed as a key and potent mechanism for disease modification in Alzheimer’s disease (AD) [1]. In addition, it is generally agreed that patients with dementia due to AD may be too far advanced in their disease to be amenable to therapeutic interventions that are expected to delay progression rather than halt neurodegeneration. Patients who are in an early stage of AD without cognitive symptoms, termed *preclinical AD*, or with mild cognitive impairment (MCI) due to AD, termed *MCI due to AD*, are seen as being in the early (predementia) AD spectrum and considered more optimally treated with disease-modifying interventions than those with clinical signs of dementia associated with more advanced disease [2].

Positive biomarker patterns, such as low cerebrospinal fluid (CSF) Aβ_1–42_ level or increased amyloid burden on positron emission tomography (PET), are seen as strong risk factors for developing AD symptoms and can help to identify patients at risk for Alzheimer dementia. Aβ is generated from the amyloid precursor protein (APP). Cleavage by β-site APP-cleaving enzyme (BACE1, β-secretase) is the first and rate-limiting step in this process, resulting in Aβ_1–42_ and other Aβ fragments (e.g., Aβ_1–40_, Aβ_1–38_, and Aβ_1–37_) that are excreted into CSF. Aβ peptide fragments of different lengths, especially Aβ_1–42_, accumulate and form Aβ plaques between neurons in the brain and are demonstrated to be neurotoxic [3, 4].

Patients with underlying AD pathology and those in early stages of AD were shown to have higher baseline BACE1 activity in CSF than healthy control subjects [5–7]. Timmers et al. [8] reported that BACE1 CSF levels showed strong correlations to all downstream AD markers, including Aβ_1–40_, Aβ_1–42_, and markers of neurodegeneration (total tau [t-tau] and phosphorylated tau [p-tau_181p_] proteins), in healthy elderly participants.

Inhibition of the activity of the BACE enzyme has been suggested to be a priority mechanism for AD therapies. Hence, the impact of lowering all Aβ fragments in CSF is expected to be stronger the earlier treatment with a BACE inhibitor is initiated. Atabecestat is a potent brain-penetrant BACE inhibitor developed by Janssen Research & Development in collaboration with Shionogi for an oral treatment of AD by reducing production of Aβ fragments. Because Aβ_1–40_ is the most prevalent form, its reduction in plasma and CSF is the primary determinant of atabecestat activity.

In prior studies in healthy elderly and young participants, atabecestat (5–150 mg) administered once daily achieved significant and sustained reduction in plasma and CSF Aβ (both up to 95% at 90 mg daily for 14 days). As such, these results supported confirmation of target engagement of atabecestat through its central BACE1 inhibition [9].

The studies reported here provide the first proof-of-mechanism (POM) data of atabecestat in patients who have evidence of elevated levels of brain amyloid and are in the early stages of the AD continuum but do not yet experience clinical symptoms of dementia related to AD. The primary objective of this study was to demonstrate pharmacodynamic (PD) activity as POM of atabecestat in the intended target population of patients with preclinical AD and patients with MCI due to AD. A 50-mg dose was selected on the basis of steady-state (day 14) CSF Aβ_1–40_ reduction from baseline observed in healthy participants (80–90%) [9], whereas a lower dose of 10 mg was chosen on the basis of exposure-response modeling and simulation (CSF Aβ_1–40_ reduction of 50–60%). In this study, effects of repeat dosing of atabecestat on lowering Aβ_1–40_ levels in CSF and plasma were evaluated as primary evidence of its target engagement (i.e., BACE1 inhibition) in the brain. Atabecestat safety, tolerability, steady-state pharmacokinetics (PK), and extent of central nervous system (CNS) penetrance were also determined as primary endpoints.

Secondary and exploratory endpoints evaluated treatment effects on the change in CSF levels of downstream biomarkers such as Aβ fragments (Aβ_1–37_, Aβ_1–38_, and Aβ_1–42_), APP fragments (sAPPα, sAPPβ, and total sAPP), t-tau/p-tau_181p_, and BACE1. The relationship between atabecestat exposure and effects on CSF Aβ_1–40_ in early AD populations was also determined.

## Methods

### Study population and selection criteria

The study population consisted of participants recruited and screened from two separate clinical trials. The ALZ1008 Study (NCT02360657) was conducted in Japan with Japanese participants, and the ALZ1005 Study (NCT01978548) was conducted in sites across Europe (Belgium, the Netherlands, Sweden, and Spain) with Caucasian participants. Potentially eligible populations with abnormal CSF Aβ_1–42_ level (concentration below cutoff value of 600 ng/L) even when CSF tau and p-tau_181p_ protein levels were normal (*see* discussion of bioanalytical assay procedures below) included those diagnosed as clinically asymptomatic (preclinical AD) who were cognitively and functionally normal (Clinical Dementia Rating [CDR] of 0), and those diagnosed with MCI due to AD who had some limited cognitive impairment but were still functionally normal and therefore had no dementia (CDR = 0.5).

Participants were screened in accordance with a baseline four-step screening process to assess their eligibility according to defined inclusion and exclusion criteria. The screening process consisted of stepwise assessments of general health, cognitive status, cerebral magnetic resonance imaging (MRI) scan and evidence of amyloid deposition by means of a positive amyloid PET scan or low CSF Aβ_1–42_ level (*see* Additional file 1: Figure S1 and Additional file 2).

Participants who had a global CDR score higher than 0.5, diagnosed with dementia due to AD, degenerative dementia such as frontal lobe dementia, cortical basal dementia, progressive supranuclear palsy and primary progressive aphasia, dementia associated with significant Parkinsonism, diffuse Lewy body disease, and multi-infarct dementia (vascular dementia) were excluded. Participants diagnosed with primary and secondary brain tumors, genetic disorder associated with dementia, severe depression, chromosome 21 trisomy, HIV dementia, and vitamin B_12_ or folic acid deficiency were also excluded. Participants with a history of malignancy within 5 years before screening, epilepsy 10 years before screening, positive tests for hepatitis B surface antigen or hepatitis C antibody, history of drug or alcohol abuse, known allergies or hypersensitivity, or a clinically significant acute illness within 7 days prior to study drug administration were excluded.

In the ALZ1005 Study, patients diagnosed as preclinical AD, aged 60 to 85 years, or as MCI due to AD, aged 50 to 90 years, were enrolled and randomized to treatment. Japanese participants diagnosed as preclinical AD (CDR-Japanese version = 0), aged 65 to 85 years, who were amyloid-positive were enrolled and randomized in the ALZ1008 Study. Participants were considered otherwise healthy for their age with a body mass index (BMI = weight/height^2^) between 18 and 35 kg/m^2^.

### Overview of trial design

Both trials were multicenter, double-blind, placebo-controlled, randomized, multiple-dose POM studies in patients with early-stage AD. The ALZ1005 Study was conducted from December 2013 to April 2015, and the ALZ1008 Study was conducted from February to September 2015. Each study consisted of an 8-week eligibility screening period with a four-step screening phase, a 4-week double-blind treatment phase, and a follow-up visit 7 to 14 days after last dosing. The maximal study duration for a participant was 14 weeks.

Fully eligible participants who completed the four-step screening period entered the treatment phase on day 1. During screening, a CSF sample was collected for biomarker diagnosis (eligibility assessment), which served as baseline for CSF PK and PD biomarker profiling.

A predose baseline blood sample was collected for PK, biomarker profiling, and clinical safety laboratory assessments. Measurement of vital signs and a 12-lead electrocardiogram (ECG) were also performed. Within each preclinical AD or MCI due to AD study population, enrolled participants were randomized at a ratio of 1:1:1 to one of two dose levels of atabecestat (10 and 50 mg) or placebo (*n* = 6–8/treatment) and self-administered single oral daily doses of study drug for 4 weeks from day 1 to day 28. Following dosing on day 1, safety, tolerability, plasma PK, and biomarkers of atabecestat were evaluated regularly on a weekly basis (days 8, 15, 22, and 28). A follow-up MRI scan was collected between days 24 and 27. In addition, on day 28, another CSF sample was collected following last dosing for PK and biomarker profiling.

### Pharmacokinetic analysis and modeling

CSF and plasma collection and processing, as well as apolipoprotein E (*APOE*) ε4 genotyping, are described in Additional file 2. Population PK modeling from sparse PK samples collected in the ALZ1005 Study was performed to derive individual steady-state maximum plasma concentration [C_max_] and area under the concentration-time profile during the dosing interval at steady state [AUC from 0 to 24 hours postdosing]. This model was also used to derive PK/PD simulations of CSF Aβ_1–40_ reduction (described in “Pharmacokinetic/pharmacodynamic relationship” section below). The ratio between the observed CSF concentration at day 28 visit and the corresponding simulated plasma concentration at the same time point (since day 28 dose) was evaluated in order to assess the extent of penetration of atabecestat into the CNS, assuming a plasma atabecestat free fraction of 6% [9].

In the ALZ1008 Study, noncompartmental PK analysis of individual atabecestat plasma concentration-time data was performed using Phoenix® WinNonlin (version 6.2.1; Certara, Princeton, NJ, USA) to estimate PK parameters on days 1 and 28 (steady state): C_max_, time to reach maximum concentration, and systemic exposure (AUC_τ_) from area under the concentration-time profile during the dosing interval were calculated by trapezoidal summation. Total apparent clearance for day 28 was calculated as dose/AUC_τ_. In addition, derived PK parameters were determined to further explore PK of atabecestat, including apparent total body clearance after extravascular administration (CL/F) and accumulation ratios for AUC_τ_ and C_max._ The extent of penetration of atabecestat into the CNS was also determined as described above for the ALZ1005 Study.

### Pharmacodynamic biomarker profiling

Atabecestat is expected to affect different forms of Aβ and precursors through its mechanism of inhibiting activity of BACE (β-secretase) enzyme. The activity of atabecestat was determined by CSF/plasma concentration profiles of Aβ fragments (Aβ_1–37_, Aβ_1–38_, Aβ_1–40_, and Aβ_1–42_), with Aβ_1–40_ being the most abundant and primary determinant of atabecestat activity in the study population. In addition, exploratory measurements of plasma/CSF levels of APP fragments (sAPPα, sAPPβ, and total sAPP) and CSF levels of BACE1, t-tau, and p-tau were performed.

### Pharmacokinetic/pharmacodynamic relationship

A semimechanistic indirect response PK/PD model was initially developed on the basis of healthy elderly data (ALZ1002 Study) [9]. In this model, atabecestat PK was assumed to drive inhibition of Aβ_1–40_ synthesis, which can be mechanistically interpreted as the PD effect of BACE inhibition [10], as illustrated in Figure S2 (*see also* Additional file 1). This model was used to simulate the expected CSF Aβ_1–40_ reduction in the ALZ1005 Study (at 3 to 6 hours postdose at steady state, using the PK observed in this study), and the predictions were then compared with the observed individual Aβ_1–40_ reductions. All analyses were conducted with the NONMEM version 7.2.0 Users Guide (1989–2011) (ICON Development Solutions, Ellicott City, MD, USA) [11].

In the ALZ1008 Study, the effects of plasma and CSF atabecestat exposure on CSF Aβ_1–40_ reduction at 3 to 6 hours postdose at steady state were explored visually. Results of PK and CSF Aβ_1–40_ reduction from Japanese preclinical AD participants in the ALZ1008 Study were compared with Caucasians with early AD and with elderly healthy volunteers from a previously published report of an atabecestat multiple-dose study [9].

### Cognitive evaluations

For the ALZ1005 Study, the effect of atabecestat on participants’ cognitive performance was explored by CDR, Repeatable Battery for the Assessment of Neuropsychological Status (RBANS), Mini Mental State Examination (MMSE), and computerized neuropsychological test battery (CANTAB) Elect assessments at screening and on day 28 (*see* Additional file 2). When multiple assessments were performed at the same visit by independent raters, the sequential order of testing was RBANS first followed by CDR, MMSE, and CANTAB Elect, and if raters for RBANS and CDR were the same, then CDR was performed prior to RBANS.

### Safety assessments

Safety and tolerability were assessed during the study by recording adverse events (AEs), clinically significant abnormalities, clinical laboratory tests, ECG, and vital signs and by performing physical, neurological, and MRI examinations. All enrolled participants were included in the safety analysis population.

### Bioanalytical procedures

#### Analysis of atabecestat

Plasma and CSF atabecestat samples were analyzed using a scientifically validated [12], specific, and sensitive LC-MS/MS method (*see* Additional file 2). The lower limit of quantification (LLOQ) was 1 ng/ml.

#### Analysis of plasma and CSF Aβ concentrations (four-plex assay)

A qualified prototype multiplex immunoassay based on Meso Scale Discovery (MSD) (Gaithersburg, MD, USA) electrochemiluminescence (ECL) detection technology was used for simultaneous detection of four Aβ species (Aβ_1–37_, Aβ_1–38_, Aβ_1–40_, and Aβ_1–42_) as described earlier [13, 14]. Aβ concentrations were determined using a standard curve with a four-parameter logistic model with the 1/*y*^2^ weighting function. All samples from each participant were analyzed in duplicate on the same assay plate. Only mean values with replicate well coefficient of variation (CV) ≤ 20% were accepted.

#### Analysis of CSF BACE1 and sAPP concentrations

BACE1 levels in CSF were analyzed using a previously described BACE1 sandwich enzyme-linked immunosorbent assay [15] (*see* Additional file 2). The sAPPα, sAPPβ, and sAPP totals were quantified in CSF using MSD ECL detection technology as described previously (*see* Additional file 2) [8, 16]. BACE1 and sAPP levels were determined using a standard curve with four-parameter logistic model with 1/*y*^2^ weighting function. All samples from each participant were analyzed in duplicate on the same assay plate. Only mean values with replicate well CV ≤ 20% were accepted.

#### Analysis of baseline CSF Aβ_1–42_ (Innotest), p-tau_181P_, and t-tau levels

Baseline Aβ_1–42_, p-tau_181P_, and t-tau concentrations were measured using Innotest® Phospho-TAU_181P_, Innotest® hTAU Ag, and Innotest® β-AMYLOID_1–42_ (Innogenetics/Fujirebio, Ghent, Belgium) and the Luminex analytical platform (Luminex Corp., Austin, TX, USA) [17]. CSF samples from both the ALZ1005 (Caucasian) and ALZ1008 (Japanese) studies were analyzed in the same laboratory setting using the same assays and analytical platform. Diagnostic threshold CSF concentrations for AD vs. normal controls for Aβ_1–42_ were applied to the current sample set to judge the likelihood of having cerebral amyloid plaque deposition [17].

### Statistical analysis

Sample sizes for the studies were not based on formal statistical testing. Based on previous clinical data, the SD for percent reduction in CSF Aβ_1–40_ ranged from 7% to 29%. Hence, assuming an SD of 16%, the precision of the 95% CI for between-treatment difference in percent reduction of CSF Aβ_1–40_ was estimated to be 21% and 10% for minimum group sizes of 6 and 16 subjects for the ALZ1008 and ALZ1005 studies, respectively.

Treatment effect on plasma/CSF Aβ as compared with placebo was estimated by 95% CI for percent changes from baseline in each of the Aβ fragments (Aβ_1–37_, Aβ_1–38_, Aβ_1–40_, and Aβ_1–42_) in CSF and plasma. For primary and secondary biomarkers, day 28% changes from baseline, the least squares (LS) means (converted to original units), and treatment differences relative to placebo (with corresponding 95% CI) were analyzed on the basis of an analysis of covariance model that included treatment group and baseline score as a covariate. Day 28 changes from baseline in CSF tau/p-tau were summarized using descriptive statistics. Data were summarized and plotted by dose group, and exposure-response relationship was explored. The relationships between central and peripheral effects on Aβ_1–40_ of atabecestat were summarized graphically by bar plots. Variability of predose baseline parameters vs. day 28 individual CSF ratios of Aβ_1–42_ to Aβ_1–40_ across treatment groups was explored graphically. Analyses were performed on individual populations of preclinical AD and MCI due to AD as well as the combined population of early AD.

## Results

### Demographics, baseline characteristics, and disposition

Participant disposition and study completion for the ALZ1005 and ALZ1008 trials are shown in Figure S3 (*see* Additional file 1). In the European trial (ALZ1005), a total of 432 participants were screened, of whom 48 patients with early AD were eligible after full screening, and 45 were enrolled and randomized to atabecestat 50 mg (preclinical AD, *n* = 6; MCI due to AD, *n* = 10), 10 mg (preclinical AD, *n* = 5; MCI due to AD, *n* = 10), or placebo (preclinical AD, *n* = 4; MCI due to AD, *n* = 10) treatment groups. Overall, the screen success rate was 10%. As expected, a higher percentage of participants with CDR = 0.5 were biomarker-positive than of those with CDR = 0 (72% vs. 32%). In the Japanese trial (ALZ1008) overall, 233 participants were screened, of whom 18 participants with preclinical AD were equally randomized to the same atabecestat treatment groups (*n* = 6/treatment; 50 mg, 10 mg, or placebo). In both trials, all randomized participants completed the studies.

Demographic characteristics, *APOE* ε4 status, and baseline CSF concentrations of BACE1 and all amyloid downstream markers and markers of neurodegeneration are summarized in Table 1. Across both studies, more males than females were randomized to treatment (Caucasian males, 53.3%; Japanese males, 72.2%). All participants in the European study were Caucasian, with mean (SD) age and BMI of 69.1 (5.44) years and 25.8 (3.31) kg/m^2^, respectively, for those in the atabecestat group and 70.4 (6.17) years and 24.5 (3.20) kg/m^2^, respectively, for those in the placebo group. Overall, a total of 34 participants were ≥ 65 years old. The reduced age limit for the preclinical AD population was driven by low probability that participants younger than 60 years will have a positive biomarker signature without any symptoms and a higher potential for those older than 85 years to proceed to AD or have significant cognitive decline. The mean (SD) age of Japanese participants in the trial was 72.1 (3.97) years, with 15 participants (83.3%) < 75 years and 3 (16.7%) ≥ 75 years.

**Table 1:** Demographics and baseline characteristics

Baseline characteristics	Caucasian				Japanese			
	Early AD (ALZ1005)			JNJ-54861911 total	Preclinical AD (ALZ1008)			JNJ-54861911 total
	Placebo	JNJ-54861911			Placebo	JNJ-54861911		
		10 mg	50 mg			10 mg	50 mg	
No. of patients	14	15	16	31	6	6	6	12
Male sex, *n* (%)	7 (50.0)	11 (73.3)	6 (37.5)	17 (54.8)	4 (66.7)	5 (83.3)	4 (66.7)	9 (75.0)
Age years, mean (SD)	70.4 (6.17)	70.6 (4.48)	67.6 (5.99)	69.1 (5.44)	74.3 (5.24)	72.2 (3.06)	69.8 (2.14)	71.0 (2.80)
Race, Caucasian, *n* (%)	14 (100.0)	15 (100.0)	16 (100.0)	31 (100.0)	–	–	–	–
Race, Japanese, *n* (%)	–	–	–	–	6 (100.0)	6 (100.0)	6 (100.0)	12 (100.0)
Baseline height, cm, mean (SD)	167.1 (11.25)	166.8 (6.71)	164.4 (10.52)	165.6 (8.83)	158.42 (6.070)	164.00 (9.894)	159.43 (12.163)	161.72 (10.836)
Baseline BMI, kg/m^2^, mean (SD)	24.5 (3.20)	26.3 (3.26)	25.3 (3.38)	25.8 (3.31)	23.49 (3.217)	22.60 (0.925)	22.39 (0.598)	22.50 (0.751)
*APOE* ε4 carrier status, *n* (%)								
No	3 (21.4)	7 (46.7)	7 (43.7)	–	4 (66.7)	3 (50.0)	5 (83.3)	–
Yes	11 (78.6)	8 (53.3)	9 (56.3)	–	2 (33.3)	3 (50.0)	1 (16.7)	–
Baseline CSF biomarkers, *n*	14	15	16		6	6	6	
Aβ_1–40_, ng/L, mean (SD)^a^	6565.86 (2393.583)	8982.57 (3811.543)	7462.00 (2267.950)	–	11,751.50 (4858.828)	8780.17 (3964.651)	9899.83 (2399.994)	–
Aβ_1–42_, ng/L, mean (SD)^a^	426.86 (164.967)	487.67 (211.560)	538.53 (320.321)		738.00 (149.689)	583.00 (226.851)	682.50 (258.667)	–
Aβ_1–37_, ng/L, mean (SD)^a^	648.43 (259.225)	736.20 (228.065)	709.50 (165.607)		367.25 (83.112)	346.50 (55.861)	450.00 (62.386)	–
Aβ_1–38_, ng/L, mean (SD)^a^	2332.93 (684.261)	2856.00 (850.390)	2649.93 (501.609)		2725.17 (1092.247)	1956.00 (735.509)	2435.83 (522.580)	–
sAPP-α, μg/L, mean (SD)	118.54 (50.009)	130.27 (42.193)	128.81 (24.222)	–	175.00 (87.391)	119.17 (85.558)	159.50 (76.839)	–
sAPP-β, μg/L, mean (SD)	153.23 (62.674)	156.60 (52.257)	178.56 (53.678)	–	174.83 (88.529)	124.33 (82.827)	181.00 (84.024)	–
Total sAPP, μg/L, mean (SD)	784.92 (378.593)	924.87 (295.937)	887.06 (187.804)	–	1143.17 (534.634)	685.17 (340.141)	1040.00 (632.984)	–
T-tau, ng/L, mean (SD)	524.62 (231.997)	618.93 (277.427)	616.40 (350.728)	–	388.83 (297.941)	338.00 (244.530)	367.33 (65.025)	–
P-tau_181_, ng/L, mean (SD)	72.58 (30.303)	80.13 (25.368)	82.29 (26.960)	–	55.83 (35.397)	49.50 (29.623)	53.33 (7.659)	–
Aβ_1–42_, ng/L, mean (SD)^b^	415.07 (86.442)	413.80 (125.575)	361.56 (77.099)		487.17 (103.747)	504.67 (95.320)	503.17 (73.216)	

*Abbreviations: AD* Alzheimer’s disease, *APOE* Apolipoprotein E, *APP* Amyloid precursor protein, *Aβ* Amyloid-beta, *BMI* Body mass index, *CSF* Cerebrospinal fluid, *P-tau*_181_ Phosphorylated tau, *sAPP* Soluble amyloid precursor protein, *T-tau* Total tau

All individual assay values were below 600 ng/L

^a^Four-plex assay data

^b^Screening Innotest assay data

Within studies, the average baseline measures of CSF biomarker levels of Aβ_1–40_, Aβ_1–42_, APP fragments, p-tau_181p_, and t-tau at baseline were comparable in each treatment group across the early AD populations. *APOE* ε4 status was comparable among atabecestat 10 mg treatment groups within each study (ALZ1005 and ALZ1008), but the number of *APOE* ε4 carriers and noncarriers was somewhat different in the placebo and 50-mg dose groups (Table 1).

### Plasma and CSF pharmacokinetic properties

**Fig. 1:**
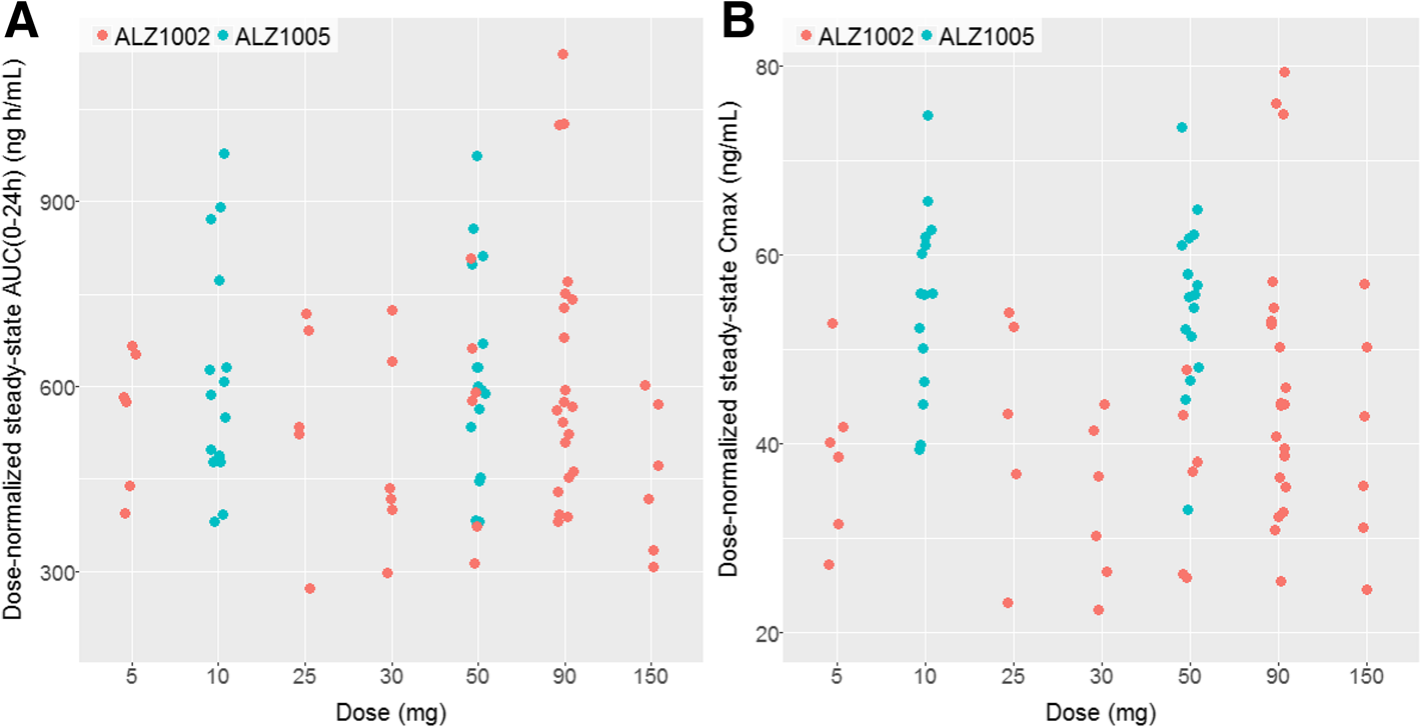
Individual atabecestat (JNJ-54861911) pharmacokinetic (PK) parameters at steady state for AUC_0–24 h_ (**a**) and maximum plasma concentration (C_max_) (**b**) vs. dose groups based on population PK model in Caucasian patients with early Alzheimer’s disease (ALZ1005) and in healthy elderly in the multiple ascending dose study (ALZ1002). Pharmacokinetic parameters were dose-normalized to 5 mg for all treatment and participant groups

The plasma PK of atabecestat after repeated daily dosing of 10 or 50 mg was adequately described by a model with linear absorption and elimination and distribution to a hypothetical peripheral compartment (i.e., two-compartment model). The linear PK of atabecestat in the ALZ1005 Study was consistent with the previous findings in healthy elderly volunteers [9]. Summary statistics of individual steady-state C_max_ and AUC_0–24 h_ by dose group are presented in Table S1 (*see* Additional file 1). Dose-normalized steady-state C_max_ and AUC_0–24 h_ were comparable to the values from the ALZ1002 Study, except for slightly higher C_max_ in Caucasians with early AD (ALZ1005) (Fig. 1).

Mean plasma concentrations of atabecestat increased with increasing dose, and no major differences in concentration-time profiles were observed between the atabecestat 10 and 50 mg groups on days 1 and 28 for Caucasian and Japanese participants and between preclinical AD and MCI due to AD groups (data not shown). In Japanese preclinical AD, mean accumulation ratios (day 28/day 1) for AUC_0–24 h_ were 165% and 177% for the atabecestat 10-mg and 50-mg groups, respectively. In Japanese participants, steady-state PK was reached at or before day 8 (based on mean predose concentrations of atabecestat at days 2, 8, 15, 22, and 28). The semilogarithmic plasma concentration plots (data not shown) showed that the concentrations in the terminal phase declined in parallel for both treatments and between participant groups on days 1 and 28.

In Caucasian patients with preclinical AD and MCI due to AD, oral clearance (CL/F) was estimated as 8.32 L/h (intersubject CV = 30%, log-normal distribution) consistent with model-based estimate from the ALZ1002 Study (10.5 L/h, CV = 19%). In Japanese patients with preclinical AD, the mean (SD) CL/F was higher (12.6 [4.96] L/h) for the atabecestat 50-mg group than for the 10-mg group (9.85 [3.51] L/h).

In the Caucasian early AD population (i.e., patients with preclinical AD and MCI due to AD), the mean (SD) CSF atabecestat concentrations at the day 28 visit for the 10-mg and 50-mg groups were 3.08 (1.04) ng/ml and 15.08 (7.07) ng/ml, respectively (*n* = 14 in each dose; one participant in each dose group did not have a sample, and one in 50-mg group had a CSF PK sample below LLOQ). In Japanese patients with preclinical AD, the corresponding CSF concentrations in the two dose groups were 3.29 (0.423) ng/ml and 19.1 (5.67) ng/ml, respectively (*see* Additional file 1: Table S1). In general, individual CSF concentrations at day 28 increased with increasing free plasma concentrations of atabecestat (sampled at nearest time point to CSF sampling on day 28).

**Fig. 2:**
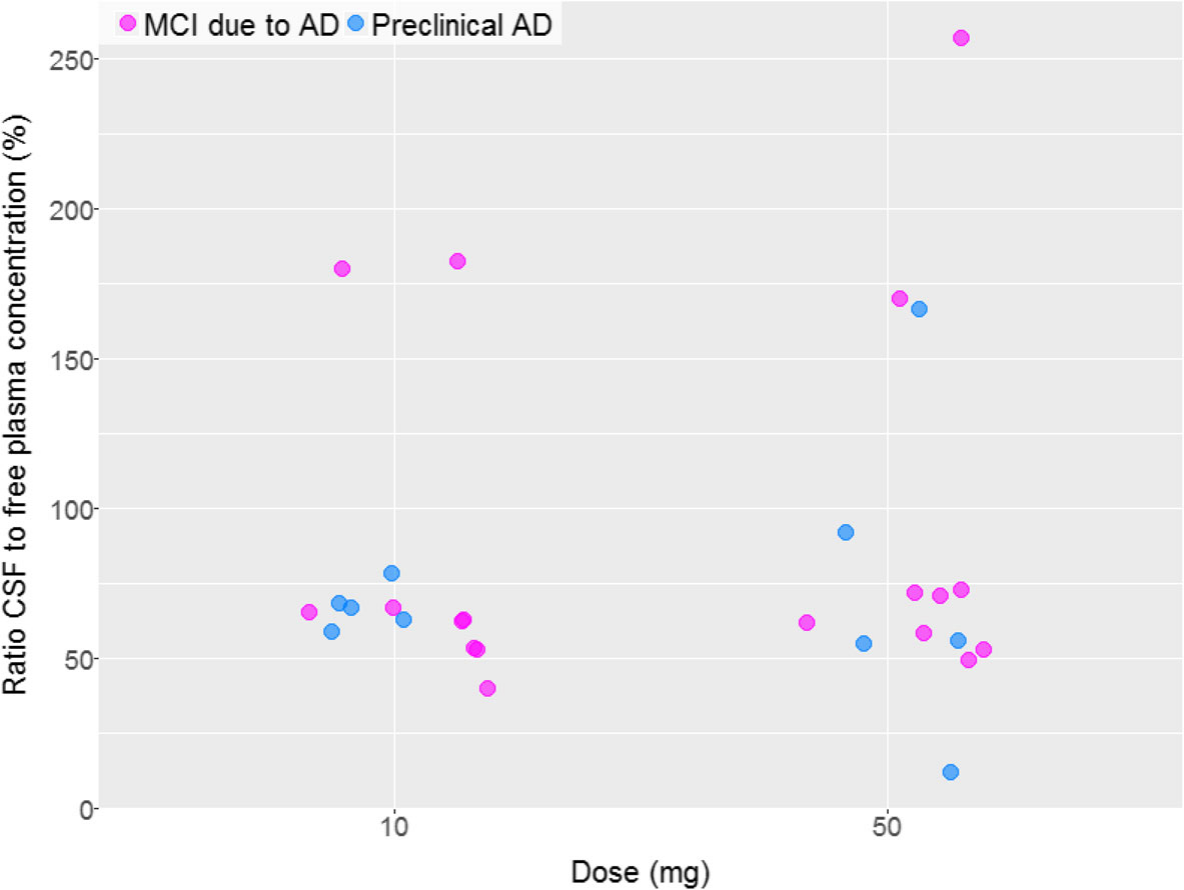
Ratio of cerebrospinal fluid (CSF) and unbound plasma atabecestat (JNJ-54861911) concentration in Caucasian patients with preclinical Alzheimer’s disease (AD) and patients with mild cognitive impairment (MCI) due to AD (ALZ1005), by dose group

In Caucasian patients with early AD, the mean ratio between CSF and free plasma atabecestat concentration was 84% with no significant differences between the 10-mg and 50-mg dose groups (Fig. 2). In Japanese, the mean ratio of CSF to free plasma concentrations at day 28 were 65% and 83% for the 10- and 50-mg groups, respectively (*see *Additional file 1: Table S1). This suggested high CNS penetrance of unbound drug at its central site of action in both Caucasian and Japanese patients with early AD.

### Pharmacodynamics of Atabecestat biomarkers

#### CSF and plasma Aβ fragments

**Fig. 3:**
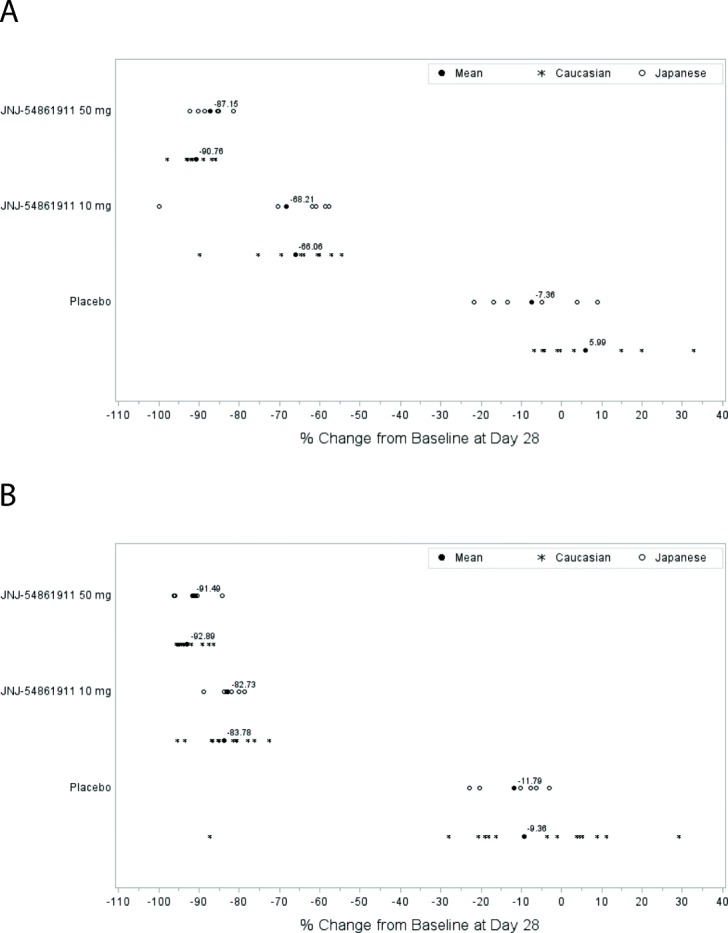
Percent change from baseline in cerebrospinal fluid (CSF) and plasma amyloid-β 1–40 (Aβ_1–40_) at day 28 (4 h postdose) across treatment groups for Caucasian patients with early Alzheimer’s disease (AD) and Japanese patients with preclinical AD. **a** Caucasian early AD and Japanese preclinical AD percent change from baseline in day 28 CSF Aβ_1–40_. **b** Caucasian early AD and Japanese preclinical AD percent change from baseline in day 28 plasma Aβ_1–40_

The primary PD endpoint was the reduction of the biomarker level of Aβ_1–40_, due to inhibition of BACE1 enzymatic synthesis by atabecestat. Figure 3 shows the individual CSF Aβ_1–40_ reductions, calculated as percent change between baseline and day 28 in each patient for the atabecestat treatment groups. The percent changes from baseline at day 28 in CSF and plasma Aβ_1–40_ and differences in LS means (SE) from placebo with 95% CI for all treatment groups are shown in Table 2. As compared with placebo, both atabecestat 10-mg and 50-mg dose groups showed large and significant reductions from baseline in CSF Aβ_1–40_ levels. Mean percent reductions for 10 mg and 50 mg vs. placebo were 67.3–89.9% vs. 3.3% in Caucasian early AD and 68.21–87.15% vs. 7.36% in Japanese preclinical AD, respectively. Similarly, there were significant percent reductions from baseline at day 28 (4 hours postdose) in plasma Aβ_1–40_ levels for atabecestat 10 mg and 50 mg as compared with placebo (Caucasian early AD, 83.8–92.9% vs. 9.4%; Japanese preclinical AD, 82.7–91.5% vs. 11.8%). The magnitude of decline as compared with placebo group was always larger in the 50-mg group than 10 mg for all participants across early AD population subtypes (Table 2). There were no meaningful differences in the magnitude of CSF and plasma Aβ_1–40_ reductions from placebo between Caucasian and Japanese populations.

**Table 2:** Percent changes in cerebrospinal fluid and plasma amyloid-β 1–40 levels from baseline at day 28 across treatment groups

Safety analysis set	Caucasian			Japanese		
	Early AD			Preclinical AD		
Percent change from baseline at day 28	Placebo	JNJ-54861911		Placebo	JNJ-54861911	
		10 mg	50 mg		10 mg	50 mg
CSF Aβ_1–40_ (ng/L), *n*	13	14	15	6	6	6
Mean (SD)	3.33 (12.389)	− 67.30 (10.602)	− 89.93 (3.535)	− 7.36 (12.122)	− 68.21 (16.174)	− 87.15 (3.898)
Median (range)	− 0.30 (− 10.3; 32.9)	− 65.22 (− 89.7; − 53.6)	− 90.66 (− 97.8; − 85.9)	− 9.18 (− 21.7; 9.0)	− 61.36 (− 99.9; − 57.7)	− 86.91 (− 92.2; − 81.5)
95% CI of mean	(− 4.16; 10.82)	(− 73.42; − 61.18)	(− 91.89; − 87.97)	(− 20.08; 5.36)	(− 85.18; − 51.23)	(− 91.24; − 83.06)
LS mean	2.73	− 66.65	− 90.01	− 6.48	− 68.96	− 87.29
Difference of LS means (SE)	–	− 69.37 (3.890)	− 92.74 (3.630)	–	− 62.48 (7.386)	− 80.81 (7.145)
95% CI	–	(− 72.25; − 61.50)	(− 100.08; − 85.39)	–	(− 78.32; − 46.64)	(− 96.13; − 65.49)
Plasma Aβ_1–40_ (ng/L) (4 hours postdose), *n*	14	15	16	6	6	6
Mean (SD)	− 9.36 (27.288)	− 83.78 (6.022)	− 92.89 (3.117)	− 11.79 (8.047)	− 82.73 (3.508)	− 91.49 (4.403)
Median (range)	− 2.37 (− 87.3; 29.2)	− 84.83 (− 95.3; − 72.4)	− 94.13 (− 95.6; − 86.3)	− 8.98 (− 23.0; −3.2)	− 82.48 (− 88.8; − 78.7)	− 91.01 (− 96.3; − 84.3)
95% CI of mean	(− 25.12; 6.39)	(− 87.11; − 80.44)	(− 94.55; − 91.23)	(− 20.24; − 3.35)	(− 86.41; − 79.05)	(− 96.11; − 86.87)
LS mean	− 9.65	− 83.45	− 92.95	− 11.79	− 82.73	− 91.49
Difference of LS means (SE)		− 73.80 (6.058)	− 83.30 (5.820)	–	− 70.94 (3.394)	− 79.70 (3.390)
95% CI		(− 86.04; − 61.57)	(− 95.06; − 71.55)	–	(− 78.22; − 63.66)	(− 86.97; − 72.43)

*Abbreviations: AD* Alzheimer’s disease, *Aβ* Amyloid-beta, *CSF* Cerebrospinal fluid, *LS* Least squares

Table 3 shows secondary endpoints for the percent reductions at day 28 from baseline for Aβ_1–37_, Aβ_1–38_, and Aβ_1–42_ fragments in CSF and their mean differences from the placebo group for all treatment groups. Both atabecestat dose groups showed greater reductions in the Aβ fragment levels as compared with the placebo group. The magnitude of the reduction was similar across the Aβ_1–37_, Aβ_1–38_, and Aβ_1–42_ fragments and consistent with the observed reductions for Aβ_1–40_ (*see* Additional file 1: Figure S4). Similarly, the magnitude of the reductions in the CSF Aβ fragment levels as compared with placebo was larger in the 50-mg dose group than in the 10-mg dose group, regardless of Aβ fragment species and patient ethnicity.

**Table 3:** Percent changes in cerebrospinal fluid amyloid-β fragment levels from baseline at day 28 across treatment groups

Safety analysis set	Caucasian			Japanese		
	Early AD			Preclinical AD		
Percent change from baseline at day 28	Placebo	JNJ-54861911		Placebo	JNJ-54861911	
		10 mg	50 mg		10 mg	50 mg
CSF Aβ_1–37_ (ng/L), *n*	12	15	14	4	2	3
Mean (SD)	0.16 (10.753)	− 66.87 (6.851)	− 95.10 (4.501)	− 19.45 (5.529)	− 61.60 (3.741)	− 89.82 (2.385)
Median (range)	0.31 (− 19.8; 19.4)	− 67.36 (− 81.1; − 52.1)	− 96.62 (− 99.4; − 87.7)	− 18.82 (− 26.2; − 14.0)	− 61.60 (− 64.2; − 59.0)	− 89.31 (− 92.4; − 87.7)
95% CI of mean	(− 6.68; 6.99)	(− 70.67; 63.08)	(− 97.70; − 92.50)	(− 28.25; − 10.65)	(− 95.22; − 27.99)	(− 95.75; − 83.90)
LS mean	0.29	− 66.94	− 95.13	− 19.89	− 62.45	− 88.67
Difference of LS means (SE)		− 67.23 (3.103)	− 95.42 (3.106)		− 42.55 (4.011)	− 68.78 (4.105)
95% CI		(− 73.52; − 60.94)	(− 101.71; − 89.12)		(− 52.86; − 32.24)	(− 79.33; − 58.23)
CSF Aβ_1–38_ (ng/L), *n*	13	15	15	6	6	6
Mean (SD)	− 5.58 (27.514)	− 58.82 (7.183)	− 87.93 (3.511)	− 10.70 (7.618)	− 62.37 (18.712)	− 83.35 (4.890)
Median (range)	− 2.21 (− 91.3; 19.2)	− 57.82 (− 76.5; − 50.9)	− 88.88 (− 91.4; − 82.4)	− 8.59 (− 21.8; − 3.0)	− 57.12 (− 99.7; − 50.4)	− 82.84 (− 88.9; − 76.1)
95% CI of mean	(− 22.20; 11.05)	(− 62.80; − 54.85)	(− 89.87; − 85.98)	(− 18.69; − 2.70)	(− 82.01; − 42.74)	(− 88.48; − 78.22)
LS mean	−7.48	− 57.34	− 87.76	− 10.21	− 62.95	− 83.26
Difference of LS means (SE)		− 49.86 (6.095)	− 80.28 (5.920)		− 52.74 (7.746)	− 73.05 (7.229)
95% CI		(− 62.19; − 37.53)	(− 92.25; − 68.31)		(− 69.35; − 36.12)	(− 88.56; − 57.55)
CSF Aβ_1–42_ (ng/L), *n*	13	15	15	6	4	6
Mean (SD)	2.36 (14.942)	− 51.86 (11.611)	− 82.48 (4.852)	− 11.79 (10.105)	− 61.22 (7.510)	− 81.51 (3.076)
Median (range)	1.08 (− 15.1; 41.7)	− 52.08 (− 72.8; − 31.2)	− 82.90 (− 90.4; − 73.5)	− 11.52 (− 26.1; 2.0)	− 60.78 (− 69.4; − 53.9)	− 81.90 (− 84.9; − 78.1)
95% CI of mean	(− 6.66; 11.39)	(− 58.29; − 45.43)	(− 85.17; − 79.79)	(− 22.39; − 1.18)	(− 73.17; − 49.27)	(− 84.74; − 78.28)
LS mean	1.40	− 51.92	− 81.58	− 11.64	− 61.45	− 81.50
Difference of LS means (SE)		− 53.32 (3.864)	− 82.97 (3.905)		− 49.81 (5.191)	− 69.86 (4.509)
95% CI		(− 61.13; − 45.50)	(− 90.87; − 75.07)		(− 61.12; − 38.50)	(− 79.69; − 60.04)

*Abbreviations: AD* Alzheimer’s disease, *Aβ* Amyloid-beta, *CSF* Cerebrospinal fluid, *LS* Least squares

In the Caucasian study population (preclinical AD and MCI due to AD), there was no meaningful difference in percent change from baseline for any of the measured Aβ species between *APOE* ε4 carriers and noncarriers across all treatment groups and AD population subtypes (*see* Additional file 1: Table S2). Given the small Japanese study population, no clear relationship could be established between *APOE* ε4 carriers and noncarriers and Aβ_1–40_ reduction in CSF and plasma.

#### CSF sAPP and t-tau/p-tau_181p_ concentrations

The percent changes from baseline in CSF APP fragment levels at day 28 and differences in LS means (SE) from placebo with 95% CI for all treatment groups are given in Table 4 and depicted graphically in Figure S5 (*see *Additional file 1). In Caucasian and Japanese patients, the CSF sAPPβ concentrations decreased after 4 weeks of treatment with both atabecestat 10-mg and 50-mg doses compared with the placebo groups vs. baseline levels. The magnitude of decrease from placebo was dose-dependent to a similar extent in both Caucasian and Japanese patients, being greater for the 50-mg groups (>-90%) compared with 10 mg groups (>-60%) (Table 4).

**Table 4:** Percent changes in cerebrospinal fluid amyloid precursor protein fragment levels and cerebrospinal fluid total tau/phosphorylated tau protein levels from baseline at day 28 across treatment groups

Safety analysis set	Caucasian			Japanese		
	Early AD			Preclinical AD		
Percent change from baseline at day 28	Placebo	JNJ-54861911		Placebo	JNJ-54861911	
		10 mg	50 mg		10 mg	50 mg
CSF sAPPα (μg/ml), *n*	12	15	15	6	6	6
Mean (SD)	− 2.60 (15.336)	88.74 (60.035)	114.28 (50.461)	11.96 (33.788)	61.37 (24.755)	82.49 (42.820)
Median (range)	− 6.14 (− 27.1; 23.2)	69.32 (28.4; 264.2)	121.38 (11.4; 187.7)	2.03 (− 21.1; 66.8)	60.22 (33.7; 104.2)	86.71 (11.6; 136.2)
95% CI of mean	(− 12.3; 7.1)	(55.5; 122.0)	(86.3; 142.2)	(− 23.50; 47.42)	(35.39; 87.35)	(37.55; 127.42)
LS mean	113.02	218.19	249.66	11.04	62.60	82.17
Difference of LS means (SE)	–	1.93 (1.100)	2.21 (1.101)	–	51.57 (21.480)	71.13 (20.649)
95% CI	–	(1.59; 2.34)	(1.82; 2.68)	–	(5.49; 97.64)	(26.84; 115.41)
CSF sAPPβ (μg/ml), *n*	12	15	15	6	6	6
Mean (SD)	− 2.05 (13.485)	− 64.34 (10.208)	− 90.70 (2.785)	12.40 (29.163)	− 66.90 (5.222)	− 91.30 (3.570)
Median (range)	− 5.32 (− 21.7; 22.7)	− 67.25 (− 75.4; − 39.6)	− 90.40 (− 96.7; − 85.3)	3.29 (− 17.5; 59.5)	− 65.78 (− 76.1; −61.1)	− 91.21 (− 96.5; − 86.0)
95% CI of mean	(− 10.6; 6.5)	(− 70.0; − 58.7)	(− 92.2; − 89.2)	(− 18.20; 43.01)	(− 72.38; − 61.42)	(− 95.05; − 87.55)
LS mean	142.68	51.70	13.51	11.98	− 65.86	− 91.91
Difference of LS means (SE)	–	0.36 (1.113)	0.09 (1.116)	–	− 77.84 (10.543)	− 103.88 (10.196)
95% CI	–	(0.29; 0.45)	(0.08; 0.12)		(− 100.45; − 55.23)	(− 125.75; − 82.02)
CSF total sAPP (μg/ml), *n*	12	15	15	6	6	6
Mean (SD)	− 7.80 (13.442)	− 13.34 (19.260)	− 15.29 (20.330)	0.06 (16.444)	− 1.55 (27.494)	− 24.85 (21.689)
Median (range)	− 5.86 (− 34.4; 19.7)	− 13.34 (− 41.3; 19.6)	− 17.39 (− 44.7; 21.9)	1.73 (− 27.0; 22.8)	− 10.40 (− 23.3; 51.2)	− 16.94 (− 53.7; − 2.6)
95% CI of mean	(− 16.3; 0.7)	(− 24.0; − 2.7)	(− 26.6; − 4.0)	(− 17.19; 17.32)	(− 30.40; 27.31)	(− 47.61; − 2.08)
LS mean	734.76	701.82	679.71	0.49	− 2.16	− 24.65
Difference of LS means (SE)	–	0.96 (1.087)	0.93 (1.086)	–	− 2.65 (14.338)	− 25.14 (13.383)
95% CI	–	(0.81; 1.13)	(0.78; 1.09)	–	(− 33.40; 28.10)	(− 53.85; 3.56)
CSF total tau (ng/L), *n*	13	15	15	6	6	6
Mean (SD)	− 1.68 (4.130)	4.18 (7.629)	8.02 (6.557)	− 17.00 (13.054)	− 4.67 (48.718)	− 38.83 (52.648)
Median (range)	− 2.48 (− 9.8; 5.6)	6.76 (− 11.2; 16.1)	7.64 (− 4.5; 23.4)	− 20.50 (− 28.0; 7.0)	− 21.00 (− 38.0; 90.0)	− 32.50 (− 110.0; 25⋅0)
95% CI of mean	(− 4.18; 0.81)	(− 0.04; 8.41)	(4.39; 11.65)	(− 30.70; − 3.30)	(− 55.79; 46.46)	(− 94.08; 16.42)
CSF P-tau (ng/L), *n*	12	15	14	6	6	6
Mean (SD)	− 1.55 (4.252)	3.56 (4.797)	7.36 (3.648)	− 1.67 (2.422)	− 0.67 (5.046)	− 2.17 (6.911)
Median (range)	0.00 (− 8.0; 5.4)	3.45 (− 4.6; 11.5)	7.42 (1.6; 16.7)	− 1.00 (− 5.0; 1.0)	− 2.00 (− 5.0; 9.0)	− 0.50 (− 11.0; 5.0)
95% CI of mean	(− 4.26; 1.15)	(0.91; 6.22)	(5.25; 9.47)	(− 4.21; 0.88)	(− 5.96; 4.63)	(− 9.42; 5.09)

*Abbreviations: AD* Alzheimer’s disease, *Aβ* Amyloid-beta, *CSF* Cerebrospinal fluid, LS Least squares, *sAPP* Soluble amyloid precursor protein

This is consistent with atabecestat deactivation of β-secretase proteolytic cleavage of APP and supportive of its mode of action. In contrast, the mean percent change in CSF sAPPα level showed a dose-related increase from baseline not exceeding twofold across all treatment groups when compared with placebo (Table 4). As expected, the magnitude of decrease in CSF total sAPP level vs. baseline was small for the 10-mg group and higher for the 50-mg group, reflective of combined effect of atabecestat on levels of sAPPβ and sAPPα fragments.

Changes from baseline in CSF t-tau/p-tau_181p_ levels at day 28 are shown in Table 4. Similar to placebo, for atabecestat 10-mg and 50-mg doses, there were no relevant changes in either CSF t-tau or p-tau over a 4-week treatment period in both Caucasian and Japanese patient groups. In Caucasians with early AD, the CSF BACE level at day 28 showed no relevant changes (i.e., > 20%) from baseline for atabecestat 10-mg and 50-mg dose groups, respectively, as depicted in Figure S6 (*see* Additional file 1).

### Pharmacokinetic/pharmacodynamic CSF Aβ_1–40_ analyses

**Fig. 4:**
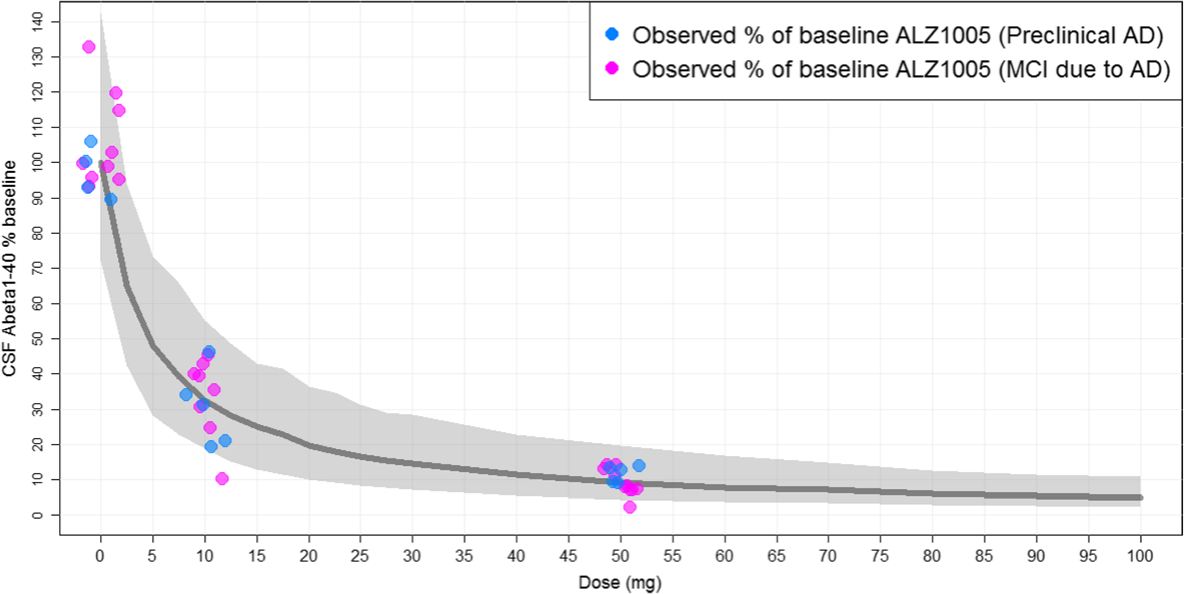
Observed day 28 cerebrospinal fluid (CSF) amyloid-β 1–40 (Aβ_1–40_) percentage reduction from baseline in Caucasian patients with preclinical Alzheimer’s disease (AD) and mild cognitive impairment (MCI) due to AD (ALZ1005) vs. model-based simulations from pharmacokinetic/pharmacodynamic modeling. The observed data of CSF Aβ_1–40_ percentage vs. baseline, stratified by patient population (preclinical AD vs. MCI due to AD) from Study ALZ1005, are overlaid on the model-predicted median and 90% prediction interval (5th and 95th percentiles, gray-shaded area) from 500 simulations per dose level

In a PK/PD model developed in healthy participants, the plasma concentration associated with 50% inhibition of Aβ_1–40_ synthesis (i.e., the potency parameter half-maximal inhibitory concentration [IC_50_]) was estimated at 21 ng/ml, and maximal inhibitory effect was fixed at 100%. Figure 4 shows the simulations obtained from the PK/PD model developed on the multiple ascending dose population and driven by atabecestat plasma PK from the ALZ1005 Study, superimposed on the actual observed CSF day 28 Aβ_1–40_ percent reductions from baseline in patients with preclinical AD and MCI due to AD for the placebo and atabecestat 10 mg and 50 mg treatment groups in the ALZ1005 Study. The new data were well in line with simulations obtained from the healthy participant PK/PD model, and therefore the potency parameter IC_50_ did not require reestimation. Baseline CSFAβ_1–40_ concentration was not a significant covariate of IC_50_ (at *p* = 0.05), which indicated that higher or lower baseline values were not associated with larger or smaller reductions from baseline. Patient population (i.e., MCI due to AD vs. preclinical AD) was not a statistically significant covariate of either IC_50_ or baseline.

The model-predicted steady-state CSF Aβ_1–40_ reduction is shown in Table 5 as median with 5th and 95th percentiles of the population for selected atabecestat doses. The observed day 28 CSF Aβ_1–40_ percent reductions in Caucasian patients with preclinical AD and MCI due to AD for both 10-mg and 50-mg groups fall well within the 5th and 95th population percentiles (Fig. 4); thus, model simulations confirmed that once-daily 10 mg and 50 mg atabecestat can attain 60–70% and 90% Aβ_1–40_ reductions, respectively. In the ALZ1008 Study, the magnitude of CSF Aβ_1–40_ reductions from baseline at day 28 generally increased with increasing concentration of atabecestat in CSF at day 28 (*see *Additional file 1: Figure S7).

**Table 5:** Steady-state cerebrospinal fluid amyloid-β 1–40 reduction from baseline at 3 to 6 hours postdose, based on population pharmacokinetic/pharmacodynamic modeling from Study ALZ1005

JNJ-54861911 dose (mg)	Steady-state CSF Aβ_1–40_ percent reductions from baseline		
	5th percentile	Median	95th percentile
5	27%	52%	72%
10	45%	67%	81%
20	63%	80%	90%
25	69%	84%	92%
30	72%	85%	93%
50	80%	91%	96%

*Aβ* Amyloid-beta, *CSF* Cerebrospinal fluid

### Cognitive effects

**Fig. 5:**
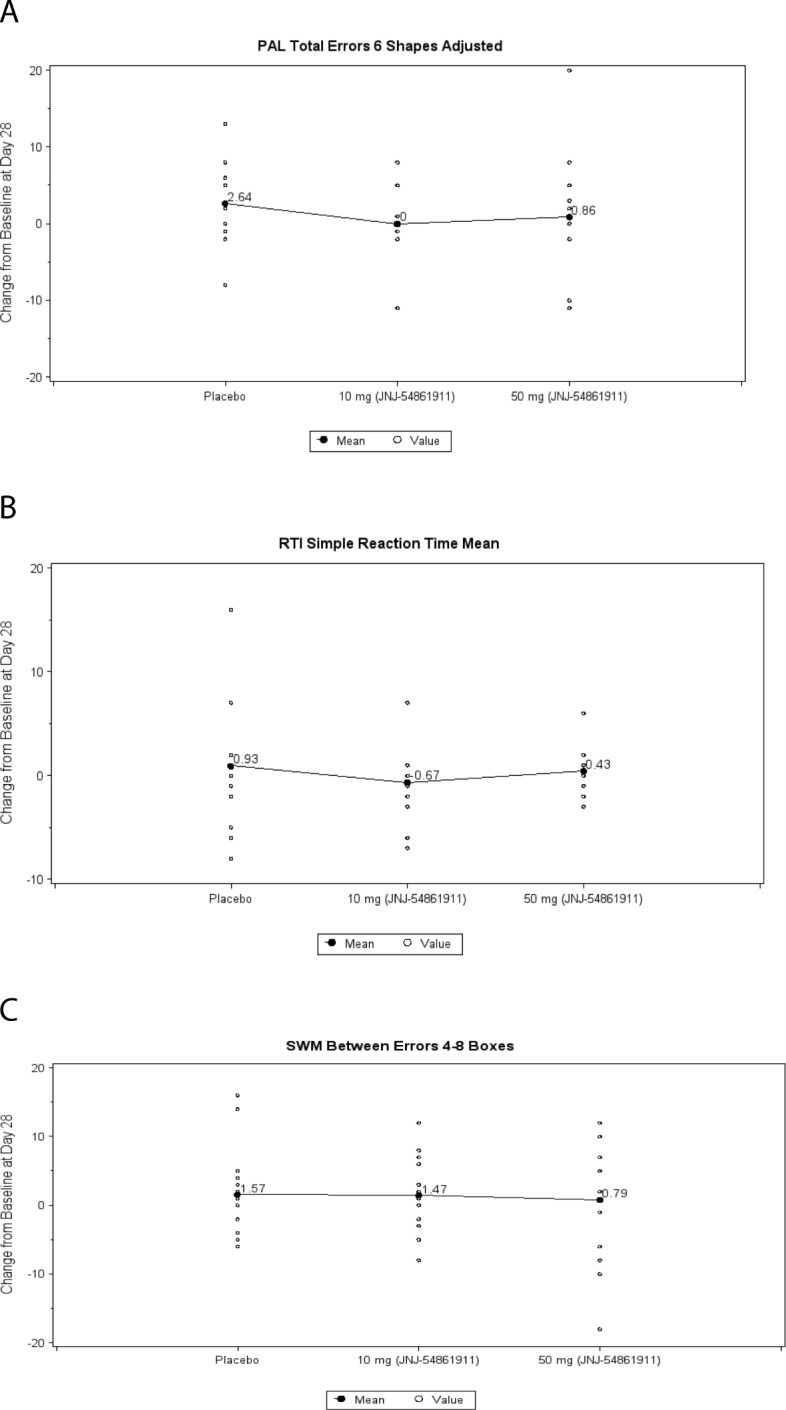
Day 28 mean change in computerized cognitive test battery elect from baseline across treatment groups in Caucasian patients with early Alzheimer’s disease (AD) (ALZ1005 safety population). **a** Paired associated learning (PAL) 6 pattern errors adjusted. **b** Reaction time (RTI) median five-choice reaction time. **c** Spatial working memory (SWM) between errors 4–8 boxes

Effect of atabecestat on cognition in Caucasians with early AD was explored to identify unexpected detrimental effects because cognitive changes were not expected over a period of 4 weeks. No meaningful changes from baseline at day 28 for all three tests (i.e., Paired Associated Learning, Reaction Time, and Spatial Working Memory) of the CANTAB outcome were found for the placebo and atabecestat treatment groups (Fig. 5). The LS mean difference of each of the atabecestat groups compared with placebo was not significant. Similarly, there was no meaningful change from baseline in day 28 RBANS total scale score, MMSE total score, and the CDR Sum of Boxes total score for any of the treatments groups (*see *Additional file 1: Table S3).

### Clinical safety

Safety data from all randomized participants who received at least one dose of study drug was included. Incidence of the treatment-emergent adverse events (TEAEs) by system organ class (SOC) in Caucasians for the ALZ1005 Study is shown in Table S4 (*see* Additional file 1). Overall, the incidence of TEAEs was low, with only 15 of 45 (33.3%) patients having one or more TEAEs during the study in which AEs occurred in 3 of 15 patients (20.0%) in the atabecestat 10-mg group, in 8 of 16 patients in 50-mg group (50.0%), and in 4 of 14 patients (28.6%) in the placebo group. The proportion of subjects experiencing TEAEs was greater in the MCI due to AD population compared with the preclinical AD population (*n* = 11/30 [36.7%] vs. *n* = 4/15 [26.7%]), with the majority of participants from the atabecestat 50-mg group.

In both atabecestat treatment groups, the most frequently reported TEAEs by SOC belonged to injury, poisoning, and procedural complications experienced by 5 of 31 patients (16.1%) that occurred in 4 patients due to post-lumbar puncture syndrome (12.9%) and in 2 patients (6.5%) due to accidental overdose. Psychiatric disorders and skin and subcutaneous tissue disorders occurred in 3 of 31 patients (9.7% each) attributed to single incidents of anxiety, depressed mood, insomnia, and irritability in the former and of dermatitis, eczema, psoriasis, and urticaria in the latter. All TEAEs were mild in severity, except for the serious adverse event (SAE) of bladder cancer, which was severe. The majority of TEAEs were considered either doubtfully related or not related to the study drug administration by the investigator. In the atabecestat 50-mg group, events that were considered to be probably related to the study drug were nausea (*n* = 1), dementia of Alzheimer’s type (*n* = 1), headache (*n* = 1), and possibly related to the study drug was urticaria (*n* = 1). These cases were not clinically relevant AEs. Only one participant receiving atabecestat 10 mg experienced a relevant abnormal neurological finding of mild ataxia observed in the knee-heel test with eyes closed and finger-nose test with eyes closed performed on day 28.

The MRI examination at the end of the treatment period showed no change in the degree of age-related white matter disease that was observed at baseline for a majority of the Caucasian study population. A small decrease from baseline in the incidence of microhemosiderin deposits in the brain was observed for participants who received both atabecestat (i.e., 90.3% had no deposits compared with 80.6% at baseline) and placebo (92.9% had no deposits vs. 71.4% at baseline).

There were no deaths or TEAEs leading to discontinuation in either study. In the ALZ1005 MCI due to AD population, there was one (*n* = 1/30; 3.3%) reported incidence of treatment-emergent SAE of bladder cancer in a 68-year-old white male patient in the placebo group. The mean and median changes over time from average predose values in ECG parameters of clinical relevance showed no treatment- or dose-related changes in the early AD population following atabecestat or placebo dosing.

During the ALZ1008 Study, there was no reported incidence of SAE in Japanese patients with preclinical AD. Only one TEAE (*n* = 1/18, 5.6%) occurred in a 70-year-old male patient in the atabecestat 50-mg group who had a TEAE of genital herpes 3 days after the start of the treatment. The TEAE duration was 8 days, considered moderate in intensity, and unlikely related to study drug by the investigator, and it resolved after treatment with valaciclovir hydrochloride. There were no clinically significant changes in QTcF and no changes from baseline > 30-ms prolongation that would be of concern.

In both studies, there were no clinically significant trends in changes from baseline in clinical laboratory analytes, liver function tests, vital sign measurements and neurologic and physical examinations.

## Discussion

Firm scientific evidence indicates that AD starts years before the first clinical symptoms. In a longitudinal study of participants with normal cognition, those who had elevated baseline brain amyloid performed worse on a number of cognitive outcome measures than those with normal brain amyloid level [18]. Findings of another retrospective review of data from the Alzheimer’s Disease Neuroimaging Initiative database of participants with MCI indicated that clinical trials in early AD should consider lowering threshold inclusion criteria for amyloid positivity with respect to baseline CSF Aβ_1–42_ levels [19]. These researchers showed that rates of neuronal injury, cognitive and functional decline, and temporal lobe atrophy substantially accelerated at conventional prethreshold levels for amyloid positivity and may have contributed to lack of efficacy in late-phase clinical trials of antiamyloid therapies [20, 21]. However, these findings point to the need for development of preventative therapies and strategies that may delay clinical symptoms of dementia in persons with AD. Investigation of biomarkers of early-stage AD pathophysiology and using PET scans for amyloid burden may allow clinical trial researchers to study asymptomatic individuals who are at risk and track their disease progression.

Atabecestat is an oral BACE1 inhibitor that was investigated in phase II/III global clinical development for early-stage AD to intercept Alzheimer’s dementia in both sporadic and genetic forms of AD. It blocks the first and rate-limiting proteolytic cleavage of APP at the β-secretase site (i.e., BACE1) that resulted in 50–80% reduction in production of the highly aggregating and neurotoxic Aβ_1–42_ species abundant in extracellular amyloid plaques [9, 22]. Since the late 2000s, other orally administered small-molecule BACE1 inhibitors with measurable brain penetration properties entered clinical trials. Among these, verubecestat (MK-8931; Merck, Whitehouse Station, NJ, USA) was in late-phase clinical studies in patients with mild to moderate AD (EPOCH study, NCT01739348) and with amnestic MCI (APECS study, NCT01953601) that were expected to read out between 2017 and 2019 and were terminated recently [23]. AZD-3293 (AstraZeneca, Cambridge, UK; and Eli Lilly and Co., Indianapolis, IN, USA) was evaluated in phase II/III trials (AMARANTH NCT02245737, DAYBREAK NCT02783573) for MCI due to AD or mild AD dementia for changes from baseline on primary and secondary outcome measures of cognition and function and was expected to report its findings by 2021, but the study was recently terminated.

This study in a patient population in the early stage of AD that has not routinely been included in AD studies is the first POM study of atabecestat that evaluated treatment effects on Aβ generation in Caucasian and Japanese cohorts diagnosed with either preclinical AD (Caucasian and Japanese, CDR/CDR-J = 0, clinically asymptomatic) or with MCI due to AD (Caucasian, CDR = 0.5). We noted a high screening burden for sites involved in the identification of such patients, with an overall eligibility success rate of about 10%. The high screen failures indicate possible challenges with patient enrollment in the early AD spectrum and the importance of using well-defined screening procedures and selection criteria to properly identify such eligible patients.

The primary endpoint showed that a 4-week treatment period with both atabecestat 10-mg and 50-mg doses as compared with placebo led to significant reductions in CSF Aβ_1–40_ (range, 67–89%) and plasma levels (range, 82–92%) and a substantial reduction in all Aβ species (Aβ_1–37_, Aβ_1–38_, Aβ_1–42_) in CSF in the study population subtypes. These results and high CNS penetrance of unbound atabecestat were similar to the previously reported findings in healthy elderly population and provide support for its POM of BACE1 inhibition in the brain in the intended target population, confirming the models developed for dose finding in ongoing clinical trials. Furthermore, agreement between PK/PD model predictions of CSF Aβ_1–40_ reductions vs. baseline and observed CSF data indicate its potential for predicting therapeutic doses from such a dose-response curve. Reported dose-related changes in CSF levels for APP fragments and magnitude and direction of change from baseline were also consistent with the atabecestat mode of action, which inhibits cleavage of APP by β-secretase confirmed in the early-stage AD patient populations studied here. Increase in CSF sAPPα from baseline was consistent with its production from subsequent post-translational processing of APP molecules cleaved by nonamyloidogenic α-secretase that is not deactivated by atabecestat.

This study was designed as a short-term POM study in a limited number of patients with early-stage AD. Thus, changes from baseline in cognitive and functional outcome measures of MMSE, CDR, and RBANS battery total scores in a Caucasian cohort were exploratory and not evaluated as formal clinical endpoints. No meaningful changes in cognitive and functional test scores were found, and all were minimally impacted for placebo and atabecestat treatment groups, indicating the absence of an apparent negative effect on cognition, which was expected due to the short treatment duration.

There were no new safety concerns in the Caucasian and Japanese early AD cohorts as compared with healthy older volunteers in the atabecestat 10-mg and 50-mg treatment groups. As compared with the placebo group, atabecestat was well tolerated during this short-term study, and there were no AEs leading to discontinuation throughout the study period. However, a trend toward higher incidence of AEs was seen in the Caucasian atabecestat 50-mg dose group, with some single AEs that were considered drug-related. The majority of AEs had resolved by end of the study.

## Conclusions

The atabecestat 10-mg and 50-mg groups showed reductions from baseline in the CSF and plasma Aβ_1–40_ levels and other Aβ fragments (Aβ_1–37_, Aβ_1–38_, and Aβ_1–42_) in CSF as compared with placebo in all population subtypes. PK/PD model simulations confirmed that once-daily 10 mg and 50 mg atabecestat can attain 60–70% and 90% Aβ_1–40_ reductions, respectively, which are considered representative for all tested Aβ fragments or species. These data confirmed the earlier reported modeling and allow prediction of Aβ fragment reduction for atabecestat doses between 5 mg and 90 mg, independent of the disease stages tested or the population. The CSF sAPPβ level showed reduction from baseline in both atabecestat dose groups as compared with placebo across AD population subtypes, whereas CSF sAPPα level increased in both doses as compared with placebo. There were no relevant changes in either CSF t-tau or p-tau_181p_ over a 4-week treatment period. Overall, atabecestat was well tolerated, the incidence of TEAEs in both studies was not meaningfully different from placebo, and no new safety signal was identified.

## Additional files

Additional file 1: **Figure S1.** Four-step screening process targeting biomarker-positive patients either as preclinical AD (CDR = 0) or with MCI due to AD (CDR = 0.5). **Figure S2.** Schematic of atabecestat (JNJ-54861911) population PK model and PK/PD model of CSF Aβ_1–40_. **Figure S3.** Consolidated Standards of Reporting Trials (CONSORT) diagram. **Figure S4.** Percent changes from baseline in CSF Aβ fragments (Aβ_1–37_, Aβ_1–38_, Aβ_1–40_, Aβ_1–42_) levels at day 28 for Caucasian (**a**) and Japanese (**b**) patients across atabecestat (JNJ-54861911) dose groups. **Figure S5.** Percent changes from baseline in CSF total sAPP level and sAPP-α and sAPP-β fragments at day 28 for Caucasian (**a**) and Japanese (**b**) patients across atabecestat (JNJ-54861911) dose groups. **Figure S6.** Percent changes from baseline in CSF BACE protein level at day 28 for Caucasian patients with early AD across atabecestat (JNJ-54861911) dose groups. **Figure S7.** Scatterplot of CSF Aβ_1–40_ reduction vs. CSF concentration of atabecestat (JNJ-54861911) after administration of 10- and 50-mg doses in Japanese preclinical AD (day 28, Study ALZ1008). **Table S1.** Atabecestat (JNJ-54861911) plasma and CSF pharmacokinetic parameters. **Table S2.** Percent reductions from baseline in CSF Aβ levels by *APOE *ε4 subgroups for early AD Caucasian population. **Table S3.** Summary of change from baseline in cognitive outcome measurements for early AD Caucasian population. **Table S4.** Incidence of treatment-emergent adverse events by body system, preferred term, and atabecestat (JNJ-54861911) treatment group for Caucasian ALZ1005 population (safety analysis set). (DOCX 374 kb)

Additional file 2: Supplementary information on the methods. (DOCX 43 kb)

## Abbreviations

AD: Alzheimer’s disease
AE: Adverse event
APOE: Apolipoprotein E
APP: Amyloid precursor protein
Aβ: Amyloid-beta
BACE: β-Secretase enzyme
BMI: Body mass index
CANTAB: Computerized neuropsychological test battery
CDR: Clinical Dementia Rating
CL/F: Oral clearance
CNS: Central nervous system
CSF: Cerebrospinal fluid
CV: Coefficient of variation
ECG: Electrocardiogram
ECL: Electrochemiluminescence
IC_50_: Half-maximal inhibitory concentration
LLOQ: Lower limit of quantification
LS: Least squares
MCI: Mild cognitive impairment
MMSE: Mini Mental State Examination
MRI: Magnetic resonance imaging
MSD: Meso Scale Discovery
PD: Pharmacodynamic
PET: Positron emission tomography
PK: Pharmacokinetic
POM: Proof of mechanism
p-tau: Phosphorylated tau
RBANS: Repeatable Battery for the Assessment of Neuropsychological Status
SAE: Serious adverse event
sAPP: Soluble amyloid precursor protein
SOC: System organ class
TEAE: Treatment-emergent adverse event
t-tau: Total tau
