# Supplementary material for: Pharmacodynamics of atabecestat (JNJ-54861911), an oral BACE1 inhibitor in patients with early Alzheimer’s disease: randomized, double-blind, placebo-controlled study
Source: Alzheimers Res Ther. 2018 Aug 23;10:85. doi: 10.1186/s13195-018-0415-6 (PMC6106931; doi:10.1186/s13195-018-0415-6)
Supplement: Supplementary file 1 — Figure S1. Four-step screening process targeting biomarker-positive patients either as preclinical AD (CDR = 0) or with MCI due to AD (CDR = 0.5). Figure S2. Schematic of atabecestat (JNJ-54861911) population PK model and PK/PD model of CSF Aβ1–40. Figure S3. Consolidated Standards of Reporting Trials (CONSORT) diagram. Figure S4. Percent changes from baseline in CSF Aβ fragments (Aβ1–37, Aβ1–38, Aβ1–40, Aβ1–42) levels at day 28 for Caucasian (a) and Japanese (b) patients across atabecestat (JNJ-54861911) dose groups. Figure S5. Percent changes from baseline in CSF total sAPP level and sAPP-α and sAPP-β fragments at day 28 for Caucasian (a) and Japanese (b) patients across atabecestat (JNJ-54861911) dose groups. Figure S6. Percent changes from baseline in CSF BACE protein level at day 28 for Caucasian patients with early AD across atabecestat (JNJ-54861911) dose groups. Figure S7. Scatterplot of CSF Aβ1–40 reduction vs. CSF concentration of atabecestat (JNJ-54861911) after administration of 10- and 50-mg doses in Japanese preclinical AD (day 28, Study ALZ1008). Table S1. Atabecestat (JNJ-54861911) plasma and CSF pharmacokinetic parameters. Table S2. Percent reductions from baseline in CSF Aβ levels by APOE ε4 subgroups for early AD Caucasian population. Table S3. Summary of change from baseline in cognitive outcome measurements for early AD Caucasian population. Table S4. Incidence of treatment-emergent adverse events by body system, preferred term, and atabecestat (JNJ-54861911) treatment group for Caucasian ALZ1005 population (safety analysis set). (DOCX 374 kb) [file 13195_2018_415_MOESM1_ESM.docx]

**Additional File 1: Supplementary Tables and Figures**

**Supplementary Figures**

Figure S1: Four step screening process targeting biomarker positive patients either as preclinical AD (CDR=0) or with MCI due to AD (CDR=0.5).

Figure S2: Schematic of atabecestat (JNJ-54861911) population PK model and PK/PD model of CSF Aβ_1-40_

Figure S3: Consolidated Standards of Reporting Trials (CONSORT) diagram

Figure S4: Percent changes from baseline in CSF Aβ fragments (Aβ_1–37_, Aβ_1–38_, Aβ_1–40_, Aβ_1–42_) levels at day 28 for Caucasian (**a**) and Japanese (**b**) patients across atabecestat (JNJ-54861911) dose groups.

Figure S5: Percent changes from baseline in CSF total APP level and sAPP-α and sAPP-β fragments at day 28 for Caucasian (**a**) and Japanese (**b**) patients across atabecestat (JNJ-54861911) dose groups.

Figure S6: Percent changes from baseline in CSF BACE protein level at day 28 for Caucasian patients with early AD across atabecestat (JNJ-54861911) dose groups.

Figure S7: Scatterplot of CSF Aβ_1–40_ reduction vs. CSF concentration of atabecestat (JNJ-54861911) after show administration of 10- and 50-mg doses in Japanese preclinical AD show (day 28, Study ALZ1008).

**Supplementary Tables**

Table S1 Atabecestat (JNJ-54861911) plasma and CSF pharmacokinetic parameters

Table S2 Percent reductions from baseline in CSF Aβ levels by *APOE* ε4 subgroups for early AD Caucasian population

Table S3: Summary of change from baseline in cognitive outcome measurements for early AD Caucasian population

Table S4 Incidence of treatment-emergent adverse events by body system, preferred term and atabecestat (JNJ-54861911) treatment group for Caucasian ALZ1005 population (safety analysis set)

**Figure S1: Four step screening process targeting biomarker positive patients either as preclinical AD (CDR=0) or with MCI due to AD (CDR=0.5).**

Step-III

Cerebral MRI

Step-IV

Amyloid Deposition

Step-II

Cognitive Status

Step-I

General Health

Vital Signs

ECG

Clinical Labs, etc.

Positive AD CSF biomarker pattern OR Positive Amyloid PET Scan

CDR ≤0.5

CDR-J = 0

No evidence of brain disease other than AD

**Figure S2: Schematic of atabecestat (JNJ-54861911) population PK model and PK/PD model of CSF Aβ_1-40_**


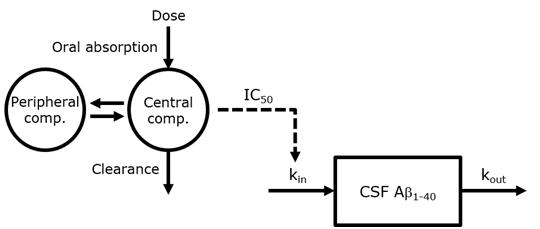


The plasma PK of JNJ-54861911 was described by a 2-compartment model with linear absorption and clearance from the central (plasma) compartment, with distribution to a hypothetical peripheral compartment. Plasma PK is assumed to drive CSF Aβ_1-40_ reduction as a result of BACE inhibition. kin: zero-order Aβ_1-40_ synthesis rate. kout: first-order Aβ_1-40_ elimination rate constant. IC50: plasma JNJ-54861911 concentration associated to 50% inhibition of Aβ_1-40_ synthesis

**Figure S3: Consolidated Standards of Reporting Trials (CONSORT) diagram**

Screened for Eligibility Caucasian (ALZ1005, N=432)

Japanese (ALZ1008, N=233)

Total Screen Failures:

Caucasian (ALZ1005)

- Not meeting inclusion criteria (n=384)

Japanese (ALZ1008)

- Not meeting inclusion criteria (n=213)

Biomarker Screening

Caucasian (N=112)
Japanese (N=126)

Eligible for Enrollment

Caucasian (N=48)

Japanese (N=20)

Not Randomized

Caucasian (n=3)

Japanese (n=2)

Randomized

Caucasian (N=45)
MCI due to AD (n=30)
Preclinical AD (n=15)
Japanese Preclinical AD (N=18)

Placebo

Caucasian MCI due to AD (n=10); Preclinical AD (n=4)

Japanese Preclinical AD (n=6)

JNJ-54861911

10 mg QD

Caucasian MCI due to AD (n=10); Preclinical AD (n=5)

Japanese Preclinical AD (n=6)

JNJ-54861911

50 mg QD

Caucasian MCI due to AD (n=10); Preclinical AD (n=6)

Japanese Preclinical AD (n=6)

Completed (100%)

Caucasian MCI due to AD (n=10);

Preclinical AD (n=4)

Japanese Preclinical AD (n=6)

Completed (100%)

Caucasian MCI due to AD (n=10);

Preclinical AD (n=5)

Japanese Preclinical AD (n=6)

Completed (100%)

Caucasian MCI due to AD (n=10); Preclinical AD (n=6)

Japanese Preclinical AD (n=6)

**Figure S4: Percent changes from baseline in CSF Aβ fragments (Aβ_1–37_, Aβ_1–38_, Aβ_1–40_, Aβ_1–42_) levels at day 28 for Caucasian (a) and Japanese (b) patients across atabecestat (JNJ-54861911) dose groups**

**A Caucasians with early AD**

**
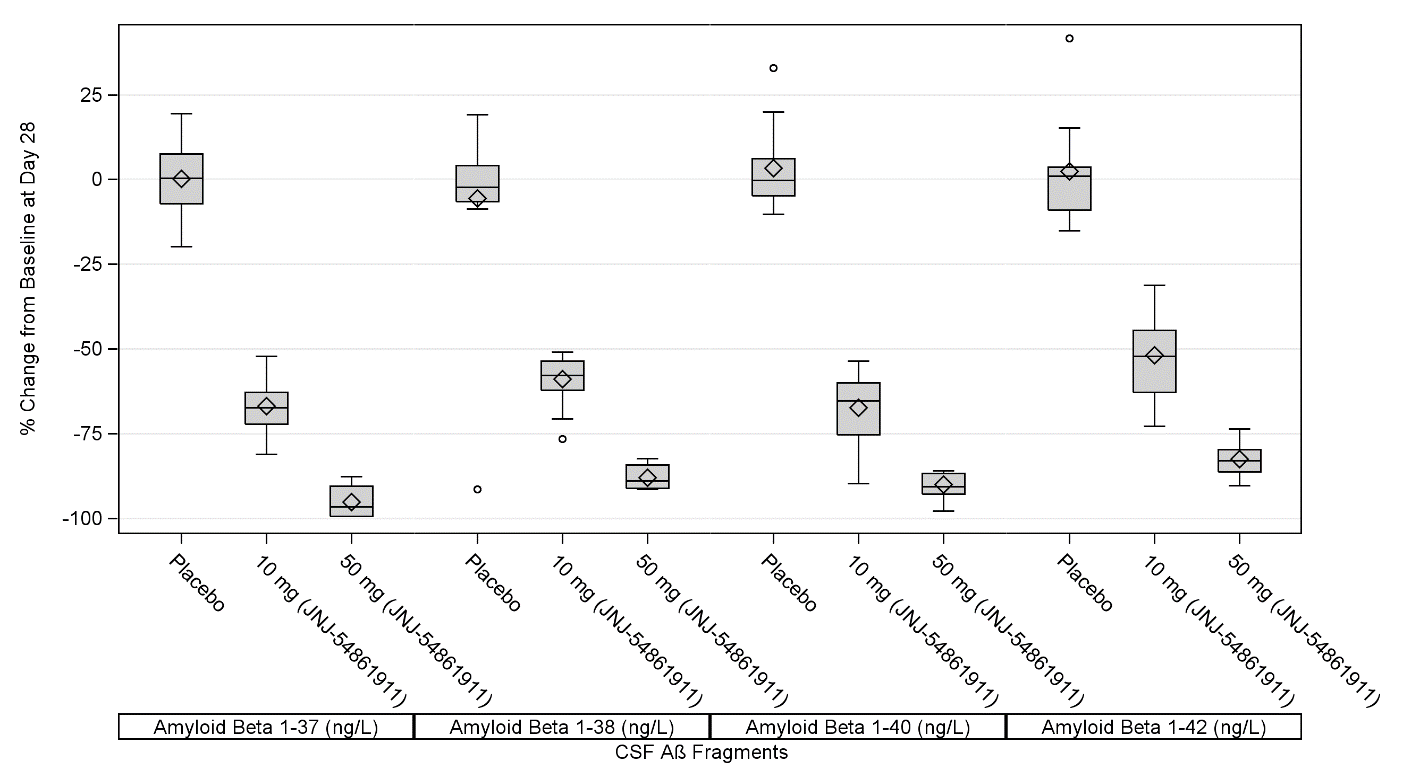
**

**B Japanese preclinical AD**


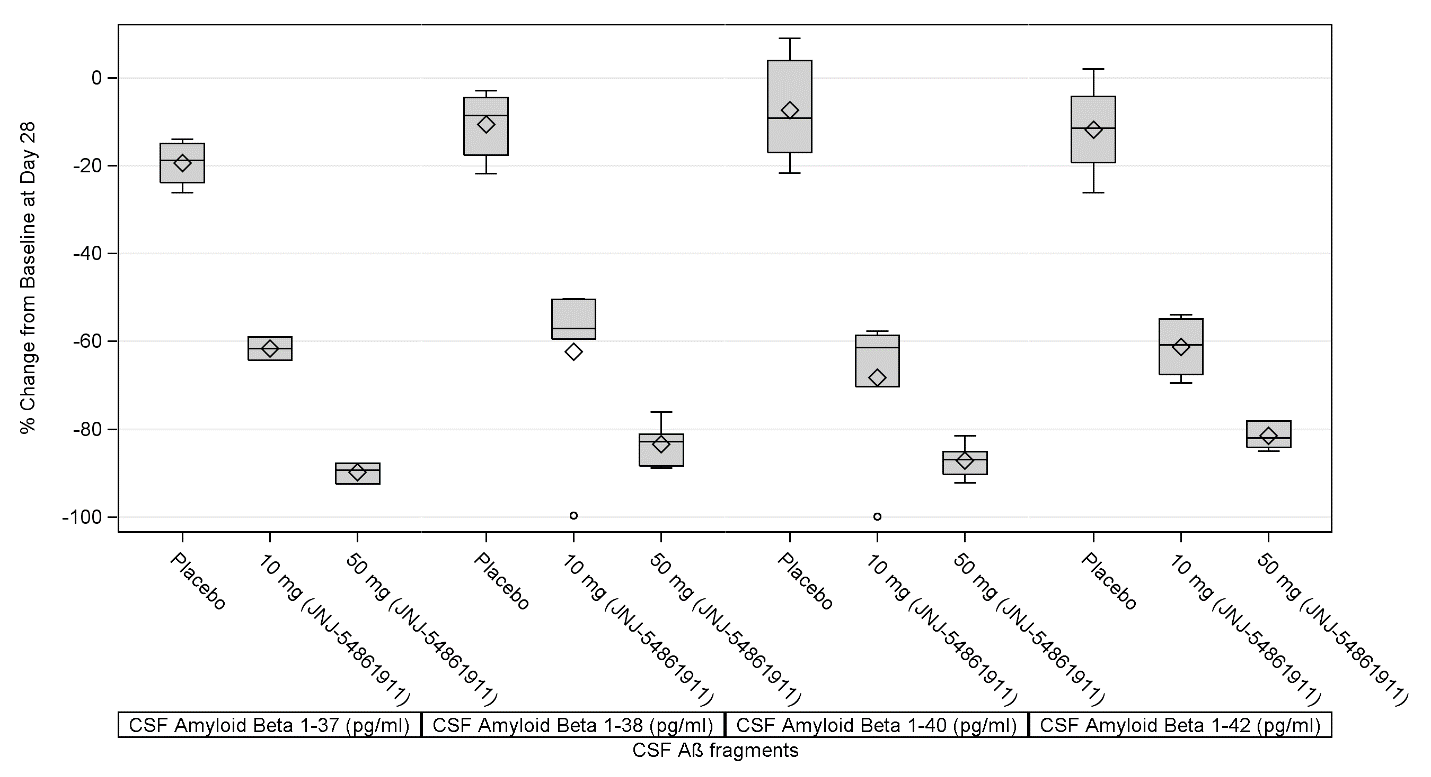


**Figure S5: Percent changes from baseline in CSF total APP level and sAPP-α and sAPP-β fragments at day 28 for Caucasian (a) and Japanese (b) patients across atabecestat (JNJ-54861911) dose groups**

**A Caucasians with early AD**


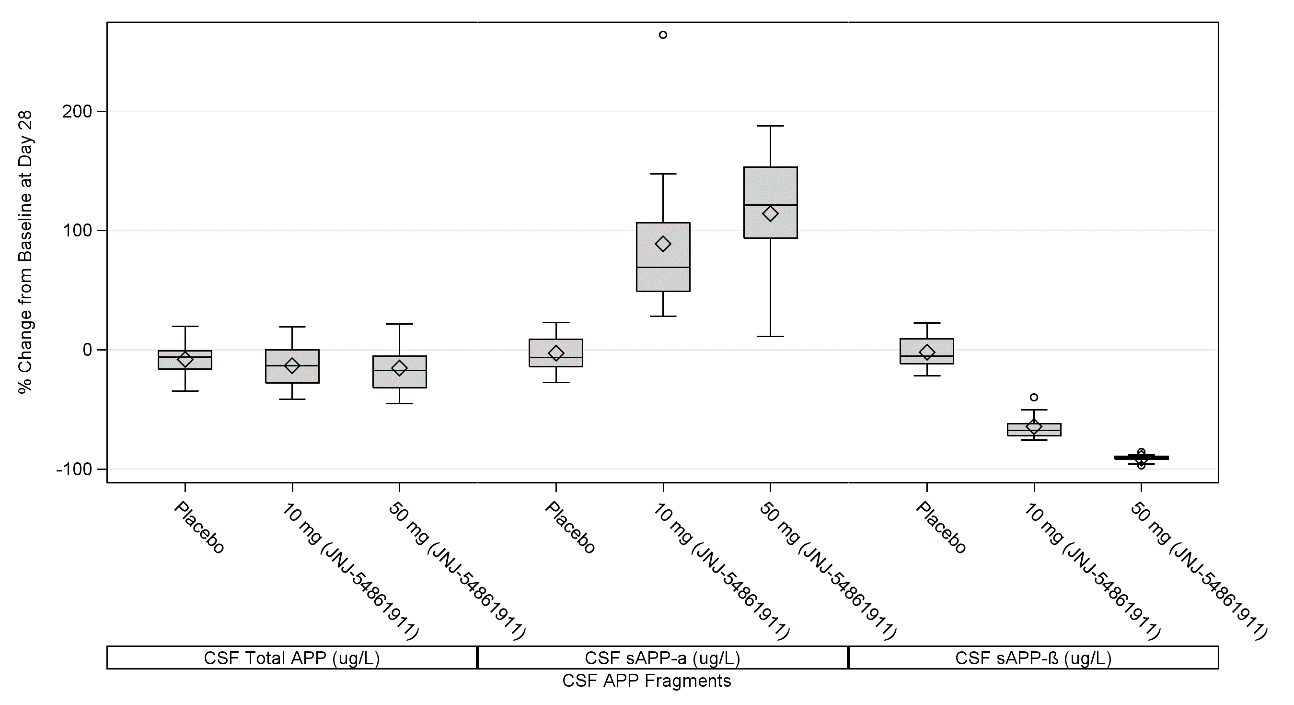


**B Japanese preclinical AD**

**
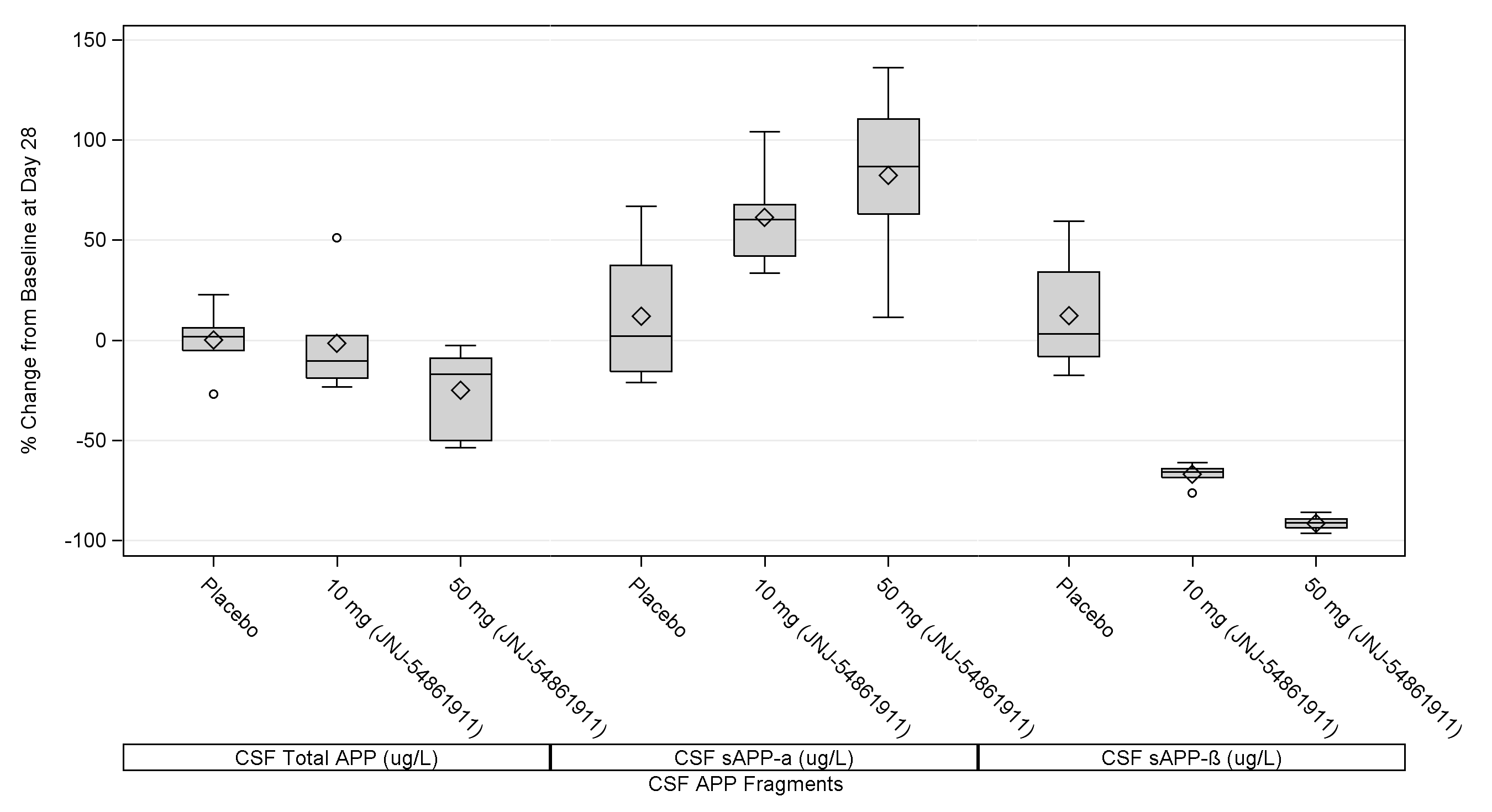
**

CSF=cerebrospinal fluid; AD=Alzheimer's disease; APP=amyloid precursor protein; sAPP-α=secreted APP-alpha; sAPP-ß=secreted APP-beta

Arrange according to above sequence (“Total” “sAPP-a” “sAPP-b”)

**Figure S6: Percent changes from baseline in CSF BACE protein level at day 28 for Caucasian patients with early AD across atabecestat (JNJ-54861911) dose groups**


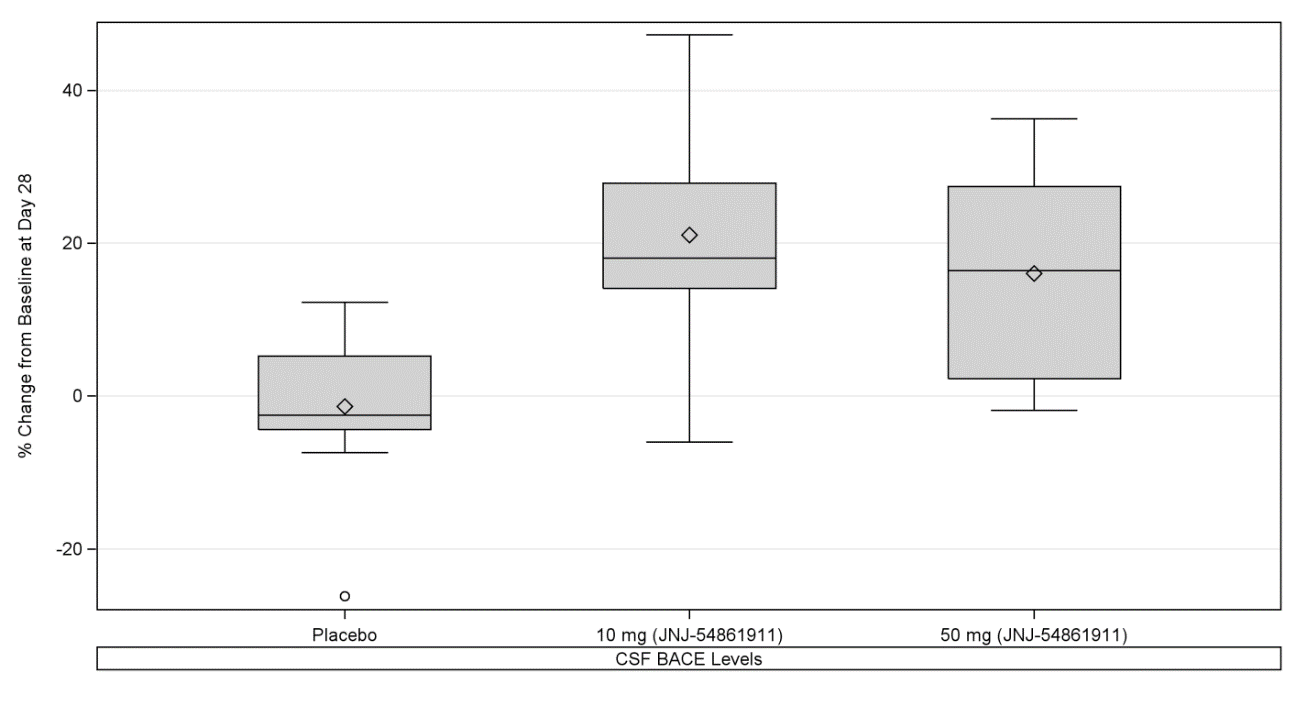


**Figure S7: Scatterplot of CSF Aβ_1–40_ reduction vs. CSF concentration of atabecestat (JNJ-54861911) after show administration of 10- and 50-mg doses in Japanese preclinical AD show (day 28, Study ALZ1008)**


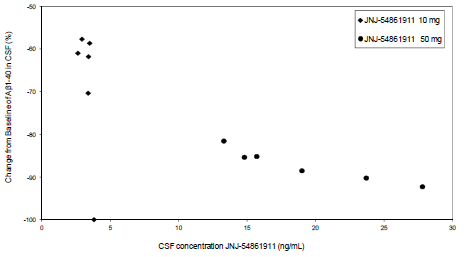


Note: one subject in the JNJ-54861911 10 mg group had CSF Aβ_1-40_ at Day 28 of 9.14 pg/mL that was below the lower limit of assay quantification (LLOQ), hence a value corresponding to 2x LLOQ was used resulting in 99.9% reduction from the subject’s baseline value (12922 pg/mL).

**Supplementary Table S1: Atabecestat (JNJ-54861911) Plasma and CSF pharmacokinetic parameters**

| **Plasma/CSF Pharmacokinetic Parameters** | **Caucasian (ALZ1005)** | | **Japanese (ALZ1008)** | |
| --- | --- | --- | --- | --- |
|  | **Early AD** | | **Preclinical AD** | |
|  | **10 mg q.d.** | **50 mg q.d.** | **10 mg q.d.** | **50 mg q.d.** |
| Plasma PK parameters, Day 28 [Mean (SD)]^a^ |  | |  | |
| N | 15 | 16 | 6 | 6 |
| T_max_^b^, h | 1.7 (0.6) | 1.8 (0.5) | 2.0 (1.0-3.0) | 1.5 (1.0-2.0) |
| C_max_, ng/mL | 110 (20) | 550 (94) | 107 (28.3) | 516 (267) |
| AUCτ, ng.h/mL | 1231 (369) | 6198 (1707) | 1106 (315) | 4729 (2547) |
| CL/F, L/h | 8.32 (30%^c^) | | 9.85 (3.51) | 12.6 (4.96) |
| Ratio C_max_, Day28/Day1, % | NC^d^ | NC^d^ | 140.39 (46.44) | 162.06 (65.05) |
| Ratio AUCτ, Day28/Day1, % | NC^d^ | NC^d^ | 165.01 (64.85) | 176.78 (83.31) |
| DN Cmax, ng/mL | 55 (10) | 55 (19) | 53.6 (14.1) | 51.6 (26.7) |
| DN AUCτ, ng.h/mL | 616 (185) | 620 (171) | 555 (158) | 473 (255) |
| CSF PK parameters, Day 28 [Mean (SD)] |  | | | |
| N | 14 | 14 | 6 | 6 |
| C_28d_, ng/mL | 3.08 (1.04) | 15.08 (7.07) | 3.29 (0.423) | 19.1 (5.67) |
| DN C_28d_, ng/mL | 1.54 (0.52) | 1.51 (0.71) | - | - |
| Ratio C_28d_/free plasma^e^, % | 78.92 (44.32) | 89.17 (64.64) | 65.00 (16.20) | 83.11 (19.30) |

^a^Estimated via population PK modeling in Study ALZ1005 due to sparse PK sampling; ^b^Median (Range); ^c^Percentage coefficient of variation based on a log-normal distribution for the inter-subject variability; ^d^Not calculated due to sparsity of PK sampling on Day 1; ^e^Calculated using the simulated plasma concentration at the same CSF sampling timepoint (ALZ1005) or the observed plasma concentration at the nearest plasma sampling timepoint to that of CSF sampling (ALZ1008), assuming a plasma free fraction of 6%. DN = dose normalization to 5 mg; C28d Median (Range), C28d, CSF=CSF concentration of JNJ-54861911 at Day 28; C28d=CSF concentration of JNJ-54861911 at Day 28

**Supplementary Table S2: Percent reductions from baseline in CSF Aβ levels by APOE ε4 subgroups for early AD Caucasian population**

| **^Pharmacogenomic Analysis Set^** | Caucasian | | |
| --- | --- | --- | --- |
|  | Early AD | | |
| **^Percent Change from Baseline at Day 28^** | **^Placebo^** | **^JNJ-54861911^** | |
|  |  | **^10 mg^** | **^50 mg^** |
| **^CSF Aβ^_1-37_ ^(ng/L)^** |  |  |  |
| **^All Subjects, N^** | ^12^ | ^15^ | ^14^ |
| **^Mean (SD)^** | ^0.16 (10.753)^ | ^-66.87 (6.851)^ | ^-95.10 (4.501)^ |
| **^Subgroup APOE ε4 =No, N^** | ^3^ | ^7^ | ^7^ |
| **^Mean (SD)^** | ^5.23 (8.660)^ | ^-70.90 (5.511)^ | ^-93.16 (4.495)^ |
| **^Subgroup APOE ε4 =Yes, N^** | ^9^ | ^8^ | ^7^ |
| **^Mean (SD)^** | ^-1.53 (11.286)^ | ^-63.35 (6.115)^ | ^-97.03 (3.870)^ |
| **^CSF Aβ^_1-40_ ^(ng/L)^** |  |  |  |
| **^All Subjects, N^** | ^13^ | ^14^ | ^15^ |
| **^Mean (SD)^** | ^3.33 (12.389)^ | ^-67.30 (10.602)^ | ^-89.93 (3.535)^ |
| **^Subgroup APOE ε4 =No, N^** | ^3^ | ^7^ | ^7^ |
| **^Mean (SD)^** | ^0.58 (5.497)^ | ^-67.58 (7.215)^ | ^-89.62 (4.477)^ |
| **^Subgroup APOE ε4 =Yes, N^** | ^10^ | ^7^ | ^8^ |
| **^Mean (SD)^** | ^4.15 (13.951)^ | ^-67.01 (13.832)^ | ^-90.20 (2.762)^ |
| **^CSF Aβ^_1-42_ ^(ng/L)^** |  |  |  |
| **^All Subjects, N^** | ^13^ | ^15^ | ^15^ |
| **^Mean (SD)^** | ^5.87 (23.710)^ | ^-34.28 (10.486)^ | ^-67.14 (8.068)^ |
| **^Subgroup APOE ε4 =No, N^** | ^3^ | ^7^ | ^7^ |
| **^Mean (SD)^** | ^7.57 (9.414)^ | ^-39.91 (10.866)^ | ^-69.48 (8.760)^ |
| **^Subgroup APOE ε4 =Yes, N^** | ^10^ | ^8^ | ^8^ |
| **^Mean (SD)^** | ^5.37 (26.993)^ | ^-29.36 (7.701)^ | ^-65.10 (7.360)^ |

**Supplementary Table S3: Summary of change from baseline in cognitive outcome measurements for early AD Caucasian population**

| **^Safety Analysis Populations^** | Caucasian | | |
| --- | --- | --- | --- |
|  | Early AD | | |
| **^Change from Baseline at Day 28^** | **^Placebo^** | **^JNJ-54861911^** | |
|  |  | **^10 mg^** | **^50 mg^** |
| **^CANTAB Elect^** |  |  |  |
| **^PAL Total Errors 6 Shapes Adjusted, N^** | ^14^ | ^15^ | ^14^ |
| **^Mean (SD)^** | ^2.64 (5.329)^ | ^0.00 (3.928)^ | ^0.86 (7.472)^ |
| **^LS Mean^** | ^2.01^ | ^0.87^ | ^0.57^ |
| **^Diff. of LS Means(SE)^** |  | ^-1.14 (2.086)^ | ^-1.44 (2.037)^ |
| **^95% CI^** |  | ^(-5.36; 3.08)^ | ^(-5.56; 2.68)^ |
| **^RTI Simple Reaction Time Mean, N^** | ^14^ | ^15^ | ^15^ |
| **^Mean (SD)^** | ^-26.79 (55.677)^ | ^11.07 (62.859)^ | ^8.28 (67.750)^ |
| **^LS Mean^** | ^-27.58^ | ^14.30^ | ^5.60^ |
| **^Diff. of LS Means(SE)^** |  | ^41.89 (23.130)^ | ^33.19 (23.367)^ |
| **^95% CI^** |  | ^(-4.90; 88.67)^ | ^(-14.08; 80.45)^ |
| **^SWM Between Errors 4-8 Boxes, N^** | ^14^ | ^15^ | ^14^ |
| **^Mean (SD)^** | ^1.57 (6.642)^ | ^1.47 (5.592)^ | ^0.79 (8.631)^ |
| **^LS Mean^** | ^1.41^ | ^1.48^ | ^0.93^ |
| **^Diff. of LS Means(SE)^** |  | ^0.06 (2.631)^ | ^-0.48 (2.693)^ |
| **^95% CI^** |  | ^(-5.26; 5.38)^ | ^(-5.93; 4.97)^ |
| **^RBANS Total Scale, N^** | ^14^ | ^15^ | ^16^ |
| **^Mean (SD)^** | ^4.29 (5.469)^ | ^0.33 (4.995)^ | ^1.88 (10.392)^ |
| **^LS Mean^** | ^4.46^ | ^0.07^ | ^1.97^ |
| **^Diff. of LS Means(SE)^** |  | ^-4.39 (2.762)^ | ^-2.48 (2.705)^ |
| **^95% CI^** |  | ^(-9.96; 1.19)^ | ^(-7.95; 2.98)^ |
| **^CDR-SB Total Score, N^** | ^14^ | ^13^ | ^14^ |
| **^Mean (SD)^** | ^0.04 (0.458)^ | ^-0.04 (0.660)^ | ^0.54 (1.082)^ |
| **^LS Mean^** | ^0.03^ | ^-0.04^ | ^0.54^ |
| **^Diff. of LS Means(SE)^** |  | ^-0.07 (0.305)^ | ^0.51 (0.300)^ |
| **^95% CI^** |  | ^(-0.69[ 0.55)^ | ^(-0.10; 1.12)^ |
| **^MMSE Total Score, N^** | ^14^ | ^15^ | ^16^ |
| **^Mean (SD)^** | ^0.21 (1.888)^ | ^-0.40 (2.971)^ | ^-0.75 (3.317)^ |
| **^LS Mean^** | ^0.57^ | ^-0.76^ | ^-0.73^ |
| **^Diff. of LS Means(SE)^** |  | ^-1.33 (0.906)^ | ^-1.29 (0.880)^ |
| **^95% CI^** |  | ^(-3.15; 0.50)^ | ^(-3.07; 0.48)^ |

^Computerized Cognitive Test Battery (CANTAB) Elect, Repeatable Battery for the Assessment of Neuropsychological Status (RBANS), Clinical Dementia Rating Scale –Sum of Boxes (CDR-SB), Mini Mental State Examination (MMSE)^

**Supplementary Table S4 Incidence of treatment-emergent adverse events by body system, preferred term and atabecestat (JNJ-54861911) treatment group for Caucasian ALZ1005 population (safety analysis set)**

|  | Caucasian | | | | | | | |
| --- | --- | --- | --- | --- | --- | --- | --- | --- |
|  | Preclinical AD | | | | MCI due to AD | | | |
|  | **^Placebo^** | **^JNJ-54861911^** | | | **^Placebo^** | **^JNJ-54861911^** | | |
|  |  | **^10 mg^** | **^50 mg^** | **^Total^** |  | **^10 mg^** | **^50 mg^** | **^Total^** |
| **^Safety Analysis Set, N^** | ^4^ | ^5^ | ^6^ | ^11^ | ^10^ | ^10^ | ^10^ | ^20^ |
| **^Subjects with any TEAE, n (%)^** | ^1 (25.0)^ | ^1 (20.0%)^ | ^2 (33.3)^ | ^3 (27.3)^ | ^3 (30.0)^ | ^2 (20.0)^ | ^6 (60.0)^ | ^8 (40.0)^ |
| **^Body System/Preferred Term^** |  |  |  |  |  |  |  |  |
| **^Gastrointestinal disorders^** | ^0^ | ^0^ | ^0^ |  | ^0^ | ^0^ | ^1 (10.0)^ | ^1 (5.0)^ |
| **^Nausea^** | ^0^ | ^0^ | ^0^ |  | ^0^ | ^0^ | ^1 (10⋅0)^ | ^1 (5.0)^ |
| **^General disorders and administration site conditions^** | ^0^ | ^0^ | ^1 (16.7)^ | ^1 (9.1)^ | ^0^ | ^0^ | ^0^ | ^0^ |
| **^Fatigue^** | ^0^ | ^0^ | ^1 (16.7)^ | ^1 (9.1)^ | ^0^ | ^0^ | ^0^ | ^0^ |
| **^Infections and infestations^** | ^0^ | ^0^ | ^0^ |  | ^1 (10.0)^ | ^0^ | ^1 (10.0)^ | ^1 (5.0)^ |
| **^Conjunctivitis^** | ^0^ | ^0^ | ^0^ |  | ^1 (10.0)^ | ^0^ | ^0^ | ^0^ |
| **^Nasopharyngitis^** | ^0^ | ^0^ | ^0^ |  | ^1 (10.0)^ | ^0^ | ^0^ | ^0^ |
| **^Vulvovaginal candidiasis^** | ^0^ | ^0^ | ^0^ |  | ^0^ | ^0^ | ^1 (10.0)^ | ^1 (5.0)^ |
| **^Injury, poisoning and procedural complications^** | ^0^ | ^0^ | ^1 (16.7)^ | ^1 (9.1)^ | ^0^ | ^2 (20.0)^ | ^2 (20.0)^ | ^4 (20.0)^ |
| **^Accidental overdose^** | ^0^ | ^0^ | ^0^ |  | ^0^ | ^1 (10.0)^ | ^1 (10.0)^ | ^2 (10.0)^ |
| **^Post lumbar puncture syndrome^** | ^0^ | ^0^ | ^1 (16.7)^ | ^1 (9.1)^ |  | ^2 (20.0)^ | ^1 (10.0)^ | ^3 (15.0)^ |
| **^Musculoskeletal and connective tissue disorders^** | ^0^ | ^0^ | ^0^ |  | ^0^ | ^0^ | ^1 (10.0)^ | ^1 (5.0)^ |
| **^Neck pain^** | ^0^ | ^0^ | ^0^ |  | ^0^ | ^0^ | ^1 (10.0)^ | ^1 (5.0)^ |
| **^Neoplasms benign, malignant and unspecified (incl cysta and polyps)^** | ^0^ | ^0^ | ^0^ |  | ^1 (10.0)^ | ^0^ | ^0^ | ^0^ |
| **^Bladder cancer^** | ^0^ | ^0^ | ^0^ |  | ^1 (10.0)^ | ^0^ | ^0^ | ^0^ |
| **^Nervous system disorders^** | ^1 (25.0)^ | ^0^ | ^0^ | ^0^ | ^1 (10.0)^ | ^0^ | ^2 (20.0)^ | ^2 (10.0)^ |
| **^Dementia Alzheimer’s type^** | ^0^ | ^0^ | ^0^ |  | ^0^ | ^0^ | ^1 (10.0)^ | ^1 (5.0)^ |
| **^Dizziness^** | ^0^ | ^0^ | ^0^ |  | ^1 (10.0)^ | ^0^ | ^0^ | ^0^ |
| **^Headache^** | ^0^ | ^0^ | ^0^ |  | ^0^ | ^0^ | ^1 (10.0)^ | ^1 (5.0)^ |
| **^Somnolence^** | ^1 (25.0)^ | ^0^ | ^0^ | ^0^ | ^0^ | ^0^ | ^0^ | ^0^ |
| **^Syncope^** | ^0^ | ^0^ | ^0^ |  | ^1 (10.0)^ | ^0^ | ^0^ | ^0^ |
| **^Psychiatric disorders^** | ^0^ | ^0^ | ^1 (16.7)^ | ^1 (9.1)^ | ^0^ | ^0^ | ^2 (20.0)^ | ^2 (10.0)^ |
| **^Anxiety^** | ^0^ | ^0^ | ^0^ |  | ^0^ | ^0^ | ^1 (10.0)^ | ^1 (5.0)^ |
| **^Depressed mood^** | ^0^ | ^0^ | ^0^ |  | ^0^ | ^0^ | ^1 (10.0)^ | ^1 (5.0)^ |
| **^Insomnia^** | ^0^ | ^0^ | ^1 (16.7)^ | ^1 (9.1)^ | ^0^ | ^0^ | ^0^ | ^0^ |
| **^Irritability^** | ^0^ | ^0^ | ^0^ |  | ^0^ | ^0^ | ^1 (10.0)^ | ^1 (5.0)^ |
| **^Renal and urinary disorders^** | ^0^ | ^0^ | ^0^ |  | ^0^ | ^0^ | ^1 (10.0)^ | ^1 (5.0)^ |
| **^Renal colic^** | ^0^ | ^0^ | ^0^ |  | ^0^ | ^0^ | ^1 (10.0)^ | ^1 (5.0)^ |
| **^Reproductive system and breast disorders^** | ^0^ | ^0^ | ^0^ |  | ^0^ | ^0^ | ^1 (10.0)^ | ^1 (5.0)^ |
| **^Vulvovaginal pruritus^** | ^0^ | ^0^ | ^0^ |  | ^0^ | ^0^ | ^1 (10.0)^ | ^1 (5.0)^ |
| **^Skin and subcutaneous tissue disorders^** | ^0^ | ^0^ | ^1 (16.7)^ | ^1 (9.1)^ | ^0^ | ^0^ | ^2 (20.0)^ | ^2 (10.0)^ |
| **^Dermatitis allergic^** | ^0^ | ^0^ | ^0^ |  | ^0^ | ^0^ | ^1 (10.0)^ | ^1 (5.0)^ |
| **^Eczema vesicular^** | ^0^ | ^0^ | ^0^ |  | ^0^ | ^0^ | ^1 (10.0)^ | ^1 (5.0)^ |
| **^Psoriasis^** | ^0^ | ^0^ | ^1 (16.7)^ | ^1 (9.1)^ | ^0^ | ^0^ | ^0^ | ^0^ |
| **^Urticaria^** | ^0^ | ^0^ | ^1 (16.7)^ | ^1 (9.1)^ | ^0^ | ^0^ | ^0^ | ^0^ |
| **^Vascular disorders^** | ^0^ | ^1 (20.0)^ | ^0^ | ^1 (9.1)^ | ^0^ | ^0^ | ^0^ | ^0^ |
| **^Hypertension^** | ^0^ | ^1 (20.0)^ | ^0^ | ^1 (9.1)^ | ^0^ | ^0^ | ^0^ | ^0^ |

Note: Incidence is based on the number of subjects experiencing at least one event, not the number of events. Adverse events are coded using MedDRA 17.0. AE=adverse events, AD=Alzheimer's disease
