# Supplementary material for: Pharmacodynamics of atabecestat (JNJ-54861911), an oral BACE1 inhibitor in patients with early Alzheimer’s disease: randomized, double-blind, placebo-controlled study
Source: Alzheimers Res Ther. 2018 Aug 23;10:85. doi: 10.1186/s13195-018-0415-6 (PMC6106931; doi:10.1186/s13195-018-0415-6)
Supplement: Supplementary file 2 — Supplementary information on the methods. (DOCX 43 kb) [file 13195_2018_415_MOESM2_ESM.docx]

**Additional file 2: Supplementary information on the Methods.**

1. Methods
   1. Baseline 4-step screening process
   2. CSF and plasma collection and processing
   3. *APOE* ε4 genotyping
   4. Analysis of atabecestat (JNJ-54861911)
   5. Analysis of BACE1
   6. Analysis of CSF sAPP concentrations
   7. Clinical evaluations
   8. References

1.1 Baseline 4-step screening process

After signing informed consent participants were screened over a period of up to 56 days to assess their eligibility according to defined inclusion and exclusion criteria. The baseline screening consisted of a 4-step process from Day-28 to Day -10. During Step I general health was assessed (vital signs, ECG and laboratory clinical tests). In step II, participant’s baseline cognitive status was graded using the CDR scale that assessed 3 domains of cognition (memory, orientation and judgment/problem solving) and 3 domains of function in structured interviews of the participant and a companion conducted by a trained rater and scored using standard methodology. Participants with a CDR of 0 were defined clinically asymptomatic in relation to cognitive deficits/dementia and those with a CDR of 0.5 were consistent with MCI due to AD, had some limited cognitive impairment but were functionally normal. Participants with a CDR global rating score >0.5 were excluded. In addition during step II, all participants performed a computerized neuropsychological test battery (CANTAB Elect) not intended as inclusion/exclusion criteria and was done to assess its validity compared with other cognitive clinical measures. This was because in ALZ1005 study initially only participants with MCI due to AD were included and their cognitive ability was assessed using CANTAB Elect. After a subsequent protocol amendment, participants who were preclinical AD were also included and their eligibility for inclusion was assessed using the CDR scale. In Step III, a cerebral MRI scan was used to exclude participants with brain disease other than potential very early signs of AD (e.g. mild hippocampal atrophy) or typical age-related changes (e.g., mild white matter hypersensitivity on MRI). The degree of white matter changes were rated on a 4-point scale and the score ranged from 0 (no lesion), 1 (focal lesions), 2 (beginning of confluent lesions), and 3 (diffuse involvement). In the last part after eligibility was conformed in Steps I-III, amyloid deposition was assessed by either evaluating baseline CSF Aβ_1-42_ level, characterized by a pattern consisting of low Aβ_1-42_ level or by an amyloid PET scan or both. During Step IV, a Mini Mental State Examination (MMSE), Repeatable Battery for the Assessment of Neuropsychological Status (RBANS) was performed to serve as a baseline predose cognitive impairment test, but not intended as study inclusion/exclusion criteria. In addition, all participants performed a second CANTAB Elect to assess test-retest reliability.

1.2 CSF and plasma collection and processing

Two CSF samples (up to 12 mL) were collected by single lumbar puncture at predose during screening and following the last study drug administration on Day 28, three to six hours post last dose. Venous blood sample (3 to 6 mL) was collected on Day 1, predose Day 2, and then weekly and at the follow-up visit (7-14 days after last dose or early withdrawal). On Day 28 serial plasma samples were collected predose until 24-hour postdose. CSF and plasma samples were collected in polypropylene tubes and aliquoted by immediate transfer of 500 µL samples to multiple storage tubes (Micronic 1.4 ml non-coded tubes U-bottom in Comorack-96, Cat No. MP22502 with caps from FluidX, Split TPE Capcluster Blue. Cat. No. 65-53028) and stored at -70°C immediately after collection. All samples analyzed in this study had at most 2 freeze-thaw cycles.

1.3 *APOE* ε4 genotyping

From all participants at predose on Day 1, a blood sample for pharmacogenomic analysis (10 mL) was collected in tubes containing potassium/sodium EDTA. DNA was isolated using Puregene chemistry and automated extraction using an Autopure LS. For all participants, *APOE* ε4 carrier status was analyzed in a multiplex reaction using polymerase chain reaction/ligation detection reaction [23]

1.4 Analysis of atabecestat (JNJ-54861911)

Atabecestat concentrations in plasma, urine and CSF were determined with a scientific validated method [16], consisting of a protein precipitation with basic methanol (0.1% diethylamine), followed by liquid chromatography coupled (reversed phase using a 5 x 2.1 mm ID XBridge Phenyl column (Waters, Milford, MA, USA), and a mobile phase of 0.1% ammonium formate pH4/methanol from 65/35 to 40/60 (v/v) running a 2-minute gradient at 0.5 mL/min) with a tandem mass spectrometer (LC-MS/MS, Sciex, Framingham, MA, USA), interfaced with a Turbo IonsprayTM in positive ionization mode. A stable isotope labeled internal standard was used for the quantification. All results were within predefined acceptance criteria. The lower limit of quantification (LLOQ) was 1 ng/mL.

The unbound fraction of atabecestat in plasma was determined by spiking the pre-dosed plasma samples from the multiple dose study with 14C-labeled atabecestat followed by ultrafiltration and radioactivity detection.

1.5 Analysis of CSF BACE1 concentration

Briefly, NUNC ninety-six-well plates (Life Technologies) were coated with 50 μl/well of capture antibody (5G7 [24]) dissolved in coating buffer (10 mM Tris-HCl, 10 mM NaCl, 10 mM NaN3, pH 8.5) with a final concentration of 2 μg/ml. After overnight incubation at 4°C, the plates were washed with PBS+0.05% Tween 20 and blocked with 100 μl/well of casein buffer (1 g casein in 1 L PBS, pH7.4) for 4h at room temperature. The coating was always done the day before the actual experiment. Samples or standards were diluted in casein buffer and mixed with the detection antibody (10B8-HRPO [24], 10mg/ml) diluted 1:2000 in casein buffer. The mixtures were added to the ELISA plates and incubated overnight at 4°C. Plates were washed and developed with 0.2 mg/ml of 3,5,3’,5’-tetramethyl-benzidine (TMB, Sigma) dissolved in 100 mM sodium acetate (NaAc, pH 4.9) supplemented with 0.03% H_2_O_2_. The reactions were allowed to proceed for maximum 15 minutes on a plate shaker at room temperature. The reactions were stopped by adding 2N H_2_SO_4_, 50 μl/well and the plates were read on a Perkin Elmer Envision 2103 multilabel reader at 450 nm. The anti-BACE1 monoclonal antibodies (mAbs) 5G7 and 10B8 were generated as described before [24]. These mAbs are highly specific for BACE1 and do not cross react with BACE2 or other structurally related aspartyl proteases [13, 24].

1.6 Analysis of sAPP CSF concentration

In brief, the assay uses P2-1 (against amino acid 104-118 of human APP695) as capturing antibody, and SULFO-TAG^TM^ labeled anti-sAPP JRD/sAPP/23, raised against the peptide sequence of amino acids 557-576 of human APP695, as detection antibody. Briefly, 96-well SECTOR^®^ standard plates were pre-wetted with PBS for 3 minutes and tapped dry, where after plates were coated with 1.25 μg/mL capture antibody overnight at 4°C. After a wash, plates were blocked and washed again. Next, 25 μL of standards or samples was applied, and the plate was incubated for 1h at room temperature on a shaker. After the next washing step, 25 μL of the detection antibody (20 μg/mL) was added per well for an additional incubation step of 1h. After the next wash step, read buffer was added to all wells, followed by 10 min of incubation. The plate was read with the Sector Imager 6000 (MSD).

1.7 Clinical Evaluations

*Clinical Dementia Rating Scale (CDR)*

The CDR assessed three domains of cognition (memory, orientation, judgment/problem solving) and three domains of function (community affairs, home/hobbies, personal care) using structured interviews of both the patient and a companion/informant by a trained rater and scored using a standard methodology. CDR global score for the six domains range from 0 to 3 with 0 indicating no dementia and 3 indicating severe dementia. It can be summed to obtain CDR Sum of Boxes (CDR-SB) with scores ranging from 0 to 18. CDR and CDR-Japanese version global score has been used in AD trials as a global measure of disease progression.

*Repeatable battery for the assessment of neuropsychological status (RBANS)*

RBANS battery includes 12 subsets that measure 5 indices; the Digit Span and Coding subtests measure Attention, the Picture Naming and Semantic Fluency subtests measure Language, the Figure Copy and Line Orientation subtests measure Visuospatial/Construction, List Learning and Store Memory subtests measure Immediate Memory, and List Recall, List Recognition, Store Memory and Figure Recall measure Delayed Memory. RBANS has been used in multinational AD clinical trials [1-3].

*Computerized Cognitive Test Battery for Screening (CANTAB Elect)*

The CANTAB test battery consists of paired associated learning (PAL), reaction time (RTI) and spatial working memory (SWM). Performance in this test battery is associated with pathologic Aβ and tau levels and enables identification of individuals with memory impairment who are likely to develop AD and those with questionable dementia [4]. Scores have a sensitivity of 0.83 and a specificity of 0.82 when differentiating individuals with MCI from healthy older adults [5]. The PAL test assesses visuospatial episodic memory and the outcome measure is the adjusted number of errors committed at the 6-pattern stage. The test-retest reliability for PAL is 0.75 in MCI and 0.85 in AD. The RTI test assesses psychomotor processing speed. The outcome measure is median duration of reaction time for executing correct trials. The test-retest reliability is 0.68 in MCI and 0.72 in AD. The SWM test assesses working memory and executive function. The outcome measure is the number errors made during performing of the task. The test-retest reliability is 0.55 in MCI and 0.68 in AD.

*Mini Mental State Examination (MMSE)*

MMSE rates subjects on orientation, registration, attention, calculation, recall and language. The maximum score is 30, and lower the score the greater the impairment.

1.8 References

1. Randolph, C., Tierney, M. C., Mohr, E., & Chase, T. N. The Repeatable Battery for the Assessment of Neuropsychological Status (RBANS): Preliminary Clinical Validity. J. Clin Exp Neuropsychol,1998 Jun; 20(3), 310-319.
2. Kotani, S., Sakaguchi, E., Warashina, S., Matsukawa, N., Ishikura, Y. et al. Dietary supplementation of arachidonic and docosahexaenoic acids improves cognitive dysfunction. Neurosci Res, 2006 Oct; 56(2), 159-164.
3. Karantzoulis S, Novitski J, Gold M, Randolph C. The Repeatable Battery for the Assessment of Neuropsychological Status (RBANS): Utility in Detection and Characterization of Mild Cognitive Impairment due to Alzheimer's Disease. Arch Clin Neuropsychol. 2013 Jul 17. PubMed PMID: 23867976.
4. Swainson R, Hodges JR, Galton CJ, Semple J, Michael A, Dunn BD, Iddon JL, Robbins TW, Sahakian BJ. Early detection and differential diagnosis of Alzheimer's disease and depression with neuropsychological tasks. Dement Geriatr Cogn Disord. 2001 Jul-Aug;12(4):265-80.
5. Chandler J. et al., 2008. Cognitive assessment: Discrimination of impairment and detection of decline in Alzheimer’s disease and mild cognitive impairment. Azheimer’s and Dementia, 4(4), pp. T551-T552
